# Computationally guided discovery of Ly6e/LY6E-dependent AAV capsid variants

**DOI:** 10.1016/j.isci.2026.115554

**Published:** 2026-04-01

**Authors:** Hiroaki Ono, Shoko Fujino, Genshiro A. Sunagawa

**Affiliations:** 1 Laboratory for Hibernation Biology, RIKEN Center for Biosystems Dynamics Research (BDR), Hyogo 651-0092, Japan; 2 Suntory Rising Stars Encouragement Program in Life Sciences (SunRiSE) Fellow, Suntory Foundation for Life Sciences, Kyoto 619-0284, Japan

**Keywords:** Drug delivery system, Neuroscience, Genetic engineering

## Abstract

Systemic adeno-associated virus (AAV) delivery to the central nervous system (CNS) would broaden neuroscience studies and therapeutic development, but capsids evolved in mice often underperform across species. We used structure-guided computational prioritization of short peptide inserts predicted to engage the BBB-associated Ly6-family receptor Ly6e/LY6E, grafting candidates into a surface loop of an AAV9 capsid. Selected variants increased blood-brain barrier transduction and enabled widespread CNS gene delivery in Syrian hamsters after intravenous dosing, with lead capsids showing Ly6e-dependent binding and transduction in cell assays. Using the same strategy for the human ortholog, we identified multiple AAV9 variants with LY6E-dependent enhancement of *in vitro* transduction. These capsids support species-matched CNS delivery and demonstrate how receptor-informed computation can focus experimental screening when *in vivo* library selection is impractical.

## Highlights

• EvoPRAISE prioritized Ly6e-binding peptide inserts for AAV9 capsid display
• Cap-PF1.7 enabled widespread CNS gene delivery in Syrian hamsters after IV dosing
• Cap-PF1.7 showed Ly6e-dependent binding and transduction in cell-based assays
• EvoPRAISE identified multiple human LY6E-dependent AAV9 variants *in vitro*

## Graphical abstract

**Figure:**
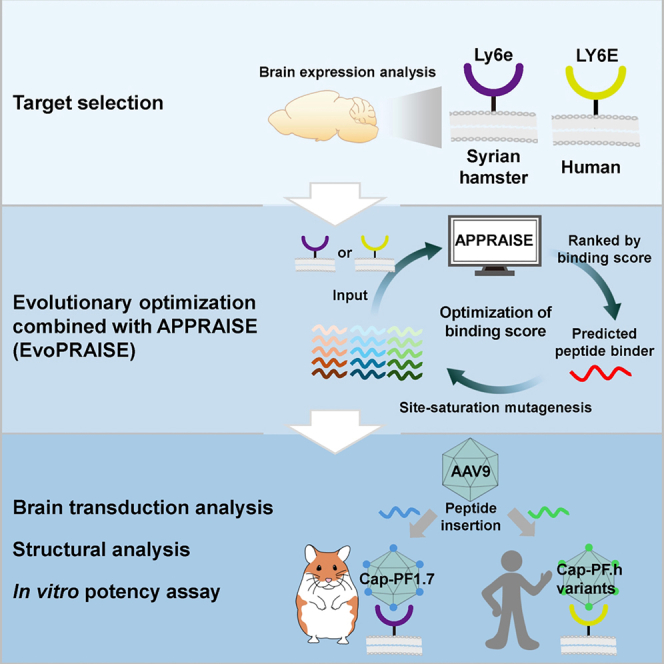

## Introduction

Recombinant adeno-associated viruses (rAAVs) are widely used as gene-delivery vectors for basic research and gene therapy. Indeed, rAAVs can transduce both dividing and non-dividing cells, persist predominantly as episomal concatemers with long-term expression, and elicit comparatively low innate and adaptive immune responses.^1^^,^^2^^,^^3^^,^^4^ rAAV genomes are compact (∼4.7 kb) single-stranded DNA molecules flanked by inverted terminal repeats, which constrains payload size but also enables modular vector design with diverse promoters and cargoes.^5^^,^^6^ Among naturally occurring serotypes, AAV9 has emerged as a versatile scaffold for engineering tissue-targeted vectors owing to its broad biodistribution after systemic administration and its proven manufacturability.^7^^,^^8^^,^^9^^,^^10^^,^^11^^,^^12^ Building on this scaffold, variable capsid-engineering strategies have produced variants with enhanced and retargeted tropism.^11^ For example, AAV-PHP.B and AAV-PHP.eB were generated to realize efficient, widespread central nervous system (CNS) transduction after intravenous delivery in mice.^10^^,^^11^ Notably, AAV.CAP-B10 was engineered to maintain strong CNS tropism in mice while minimizing off-target liver transduction.^12^

Much of modern capsid engineering relies on *in vivo* selection of highly diverse libraries (typically 10^5^–10^7^ variants) generated by peptide display (e.g., 7-mer NNK),^10^^,^^11^ error-prone mutagenesis,^13^^,^^14^ and/or DNA shuffling.^15^^,^^16^^,^^17^ After systemic administration, enriched variants are recovered from target tissues by next-generation sequencing of barcodes or capsid ORFs. Iterative rounds of this procedure yield candidates with improved performance. This directed-evolution paradigm is powerful precisely because it does not require prior knowledge of the target receptor or mechanism of entry. However, AAV variants isolated in one host frequently fail to generalize, even across mouse strains, and seldom translate to non-human primates. For example, PHP.eB crosses the blood-brain barrier (BBB) in C57BL/6 but loses activity in BALB/c, reflecting strain-specific expression of entry factors and vascular contexts.^18^^,^^19^ More broadly, rodent-selected CNS-tropic capsids have not reproduced robust BBB transduction in primates.^20^^,^^21^

These translation gaps become a major problem when a phenotype of interest spans taxa. Hibernation exemplifies this challenge. Hibernating mammals can survive prolonged periods of scarcity by markedly suppressing basal metabolic rate and lowering core body temperature to ∼10 °C, often for days or weeks.^22^^,^^23^^,^^24^ In laboratory mice, exposure to low ambient temperature and food restriction can elicit daily torpor. This condition is superficially similar to hibernation, albeit shorter and comprising a single-cycle state with distinct thermoregulatory control mechanisms.^25^^,^^26^ Recent work has identified hypothalamic neuronal populations that can drive hibernation-like hypothermia and hypometabolism in mice, implicating the CNS in the control of these states.^27^^,^^28^ Yet the extent to which these circuits and their molecular entry points are conserved in natural hibernators remains unclear. Hibernators that can be maintained in laboratory settings (e.g., Syrian hamsters) currently lack fully optimized genetic-modification toolkits. As such, AAV-mediated gene delivery is an attractive approach for CNS studies of hibernation.

In recent years, rational design has become far more accessible thanks to advances in structural biology and the accumulation of domain knowledge.^29^^,^^30^ Rational, receptor-informed approaches can complement large *in vivo* selections, particularly for non-model species and for targets where repeated rounds of animal screening are impractical. Recent work has shown that BBB-penetrant AAVs can be engineered through defined capsid-receptor interactions, enabling vectors with known mechanisms of action and more predictable tropisms.^31^^,^^32^ These studies also highlighted that several mouse-selected BBB-crossing capsids rely on LY6-family proteins, underscoring the importance of receptor context for translation beyond standard mouse models. One practical route is to graft receptor-binding modules (e.g., short peptides or nanobodies) into permissive, surface-exposed loops of the AAV capsid so that the vector engages proteins displayed by the target tissue. Progress in protein-structure prediction, including AlphaFold2/multimer, has made it increasingly feasible to model peptide-receptor interactions and use these models to prioritize candidates.^33^^,^^34^^,^^35^^,^^36^ Notably, automated pairwise peptide-receptor analysis for screening engineered proteins (APPRAISE) evaluates candidate peptide-receptor pairs by building complex models (via AlphaFold-Multimer^37^ or ESMFold^38^) and scoring them with fast, interpretable metrics that incorporate biophysical and geometric constraints.^39^^,^^40^ By considering interface complementarity and steric accessibility, APPRAISE helps filter candidates toward those most likely to be compatible with capsid display. In this study, we use APPRAISE as a structure-guided filter to prioritize Ly6e-interacting peptide candidates for capsid display and to focus subsequent experimental testing.

Here we present a Ly6e/LY6E-focused study in which receptor-informed computational prioritization and iterative refinement were used to engineer AAV9-derived capsids with enhanced BBB transduction. Focusing on the Syrian hamster—a laboratory-manageable model relevant to hibernation biology where efficient CNS gene delivery remains challenging—we prioritized short peptides predicted to interact with Ly6e, a highly expressed Ly6-family member in hamster brain, and grafted them into a permissive loop of the AAV9 capsid. This approach yielded variants that cross the BBB following systemic administration and mediate widespread CNS transduction in Syrian hamsters. We further show that transduction by lead variants is Ly6e-dependent, supporting Ly6e as an entry factor for these capsids, and we extend the design to the human ortholog LY6E to identify multiple variants with LY6E-dependent enhancement of transduction *in vitro*.

Together, our results identify Ly6e-targeting, BBB-penetrant AAV capsids for species-matched CNS delivery in the Syrian hamster and demonstrate Ly6e/LY6E-dependent transduction with computationally prioritized peptide inserts. In the Ly6e/LY6E setting, this work illustrates how receptor-informed computational prioritization can help reduce experimental screening and focus limited *in vivo* resources when large library approaches are not readily feasible.

## Results

### Computational prioritization and iterative refinement identify Ly6e-interacting peptides for AAV9 capsid display

**Figure 1:**
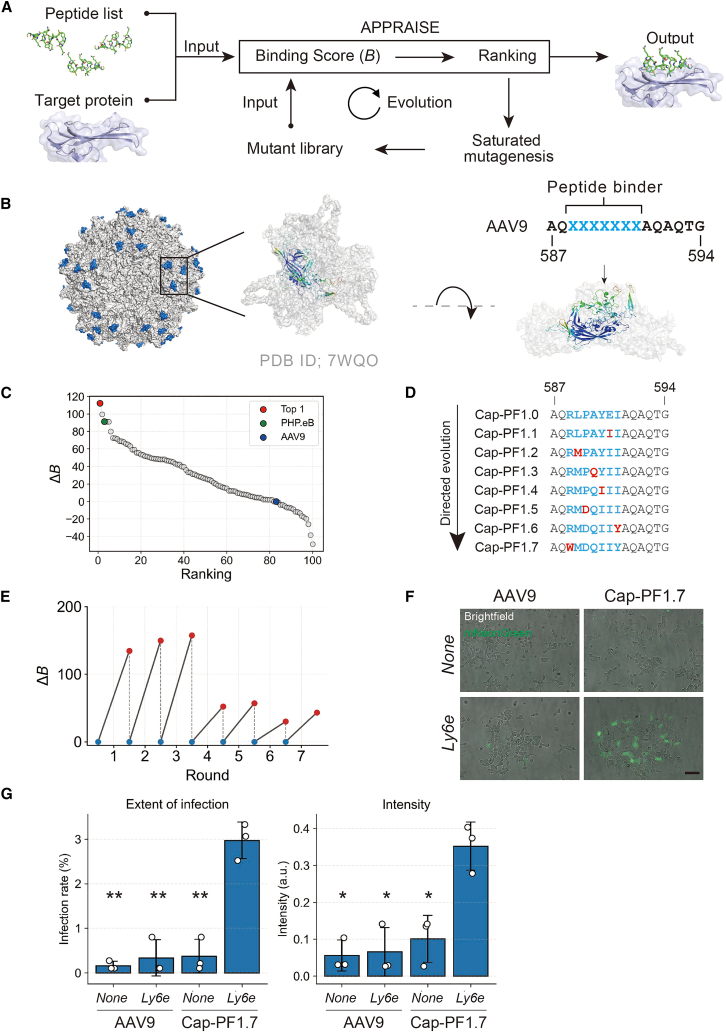
Workflow of EvoPRAISE and its application in generating a peptide binder for Ly6e (A) Workflow of EvoPRAISE. Firstly, the extracellular domain of the target membrane protein and a pool of randomly generated peptides were prepared. Secondly, these inputs were processed by APPRAISE, which calculates an energetic binding score (B) for each peptide based on atom counting. Peptides were ranked according to binding score, and the top candidate was selected (Round 0). For the top-ranked peptide, a saturation mutagenesis library was generated by substituting each residue with one of the 19 other common natural amino acids. This library was then evaluated by APPRAISE to determine a new top-ranking peptide (Round 1). In subsequent rounds, residues that had already evolved were fixed, while saturation mutagenesis was applied to the remaining residues. This iterative process was repeated until all residues had been evolved. (B) Structural model of AAV-PHP.eB, which highlights the peptide insertion site in blue. The left panel shows the AAV capsid composed of 60 structurally identical subunits (PDB ID: 7WQO). The middle panels show top views around the 3-fold symmetry axis, with the three subunits forming the trimer displayed. A single VP3 subunit is highlighted in green, and the inserted peptide sequence is shown in blue. Peptide sequence used as the EvoPRAISE input. Seven-residue peptide binders were inserted between residues 588 and 589 (VP1 numbering) in a surface-exposed variable region of AAV9. (C) Binding scores of 100 randomly generated peptides were compared with that of the AAV9 peptide (AQAQAQTG) and plotted as ΔB in ranking order. In Round 0, the RLPAYEI peptide (red) ranked first. The peptide pool also included the PHP.eB peptide (green) and the AAV9 peptide (blue). (D) Amino acid sequences at the AAV9 VP1 peptide-insertion site are shown for each variant along the directed-evolution trajectory (arrow). The 7-mer insert sequence (blue; residues 588–594, VP1 numbering) was iteratively optimized from RLPAYEI (Cap-PF1.0) to WMDQIIY (Cap-PF1.7). Red letters indicate the amino acid substitutions that emerged in that round relative to the preceding variant. Numbers denote the flanking VP1 residue positions (587 and 594). (E) Changes in binding scores of the top-ranked peptides across rounds. Red plots indicate the top peptide of each round. In the subsequent round, the same peptide was used as the reference for comparison against its variants (blue). (F) *In vitro* infectivity assay. AAV.Cap-PF1.7 showed Ly6e-dependent enhancement of transduction in HEK293T LY6E-KO cells overexpressing *Ly6e*, whereas the negative-control AAV9 did not. AAV capsids carrying a fluorescent protein expression cassette were applied at 5 × 10^9^ viral genomes (v.g.) per well to HEK293T cells transfected or not with *Ly6e* in a 96-well plate format. Images were taken 24 h after transduction (*n* = 3 per condition). Scale bars, 200 μm. (G) Bright-field and mNeonGreen images were quantified to calculate extent of infection (infection rate, %; left) and intensity (brightness per transduced area, a.u.; right) for AAV9 and Cap-PF1.7 under LY6E-KO (*None*) or LY6E-expressing (*Ly6e*) conditions. Bars represent mean ± SD; open circles denote individual image measurements. Asterisks indicate comparisons of Cap-PF1.7 under Ly6e-expressing conditions versus each of the other indicated groups (∗*p* < 0.05, ∗∗*p* < 0.01; Welch’s two-sided *t* test with Holm-Bonferroni correction).

To identify Ly6e-interacting peptides suitable for capsid display, we combined APPRAISE-based structure-guided prioritization with an iterative mutagenesis-and-selection scheme. Briefly, candidate 7-mer peptides were ranked by APPRAISE,^39^ top candidates were diversified by site-saturation mutagenesis, and the resulting variants were re-ranked to select improved candidates over successive rounds (Figure 1A). This workflow generated a stepwise trajectory of optimized peptide inserts for evaluation on an AAV9 capsid scaffold. We refer to this workflow as evolutionary optimization combined with APPRAISE (EvoPRAISE).

We applied EvoPRAISE to identify rAAV capsids capable of enhanced blood-brain barrier (BBB) transduction in the Syrian hamster. Previous large-scale *in vivo* screening studies have yielded variants such as AAV-PHP.eB^41^ and AAV.CAP-B10,^12^ which can cross the BBB and efficiently transduce the brain. These capsids are AAV9-derived variants displaying short (7 amino acid) surface peptides. Genetic and biophysical studies have shown that binding to LY6A, a GPI-anchored membrane protein, is essential for BBB penetration and CNS entry.^31^ To enable BBB transduction in the Syrian hamster, we aimed to design peptide binders for the Ly6 family expressed in the Syrian hamster brain, with the goal of generating an AAV capsid capable of transducing the CNS of the Syrian hamster. We designed 7-mer peptide binders to be inserted into residues 588–589 of the VP1 capsid protein, a surface-exposed region that can directly contact receptors in the assembled capsid^42^^,^^43^ (Figure 1B).

To determine the molecular target, we examined Ly6 family genes expressed in the Syrian hamster brain. Genome analysis (GCF_017639785.1 assembly) identified at least 18 Ly6 family genes (Figure S1A). To select the most abundantly expressed transcript, qPCR was performed using bulk brain samples, which revealed that *Ly6e* was the most highly expressed gene (Figure S1B). Consistent with its potential relevance for BBB-facing targeting, *Ly6e* expression can also be confirmed in vascular endothelial cell populations in mouse brain scRNA-seq datasets.^44^ We therefore set Ly6e as the target protein for EvoPRAISE.

As a starting point for the directed evolution, 100 random 7-mer peptides were generated along with two capsid controls (PHP.eB and wild-type AAV9). The extracellular domain of Ly6e (residues 27–118) was used as the target receptor input. APPRAISE ranked the peptides according to their binding score to Ly6e (residues 27–118). This analysis revealed the RLPAYEI peptide (Cap-PF1.0) to display the top binding score (Figure 1C). Next, directed evolution of Cap-PF1.0 was initiated. A mutant library in which each amino acid of RLPAYEI was substituted with all other 19 natural amino acids was generated. Because exhaustive evaluation of the full single-mutant library in one run would require several days of computation even on an NVIDIA V100 GPU (Google Colab), we applied APPRAISE to identify the top-ranked mutant at each amino acid position. The best candidates from all positions were then pooled, and the top-ranked peptide (RLPAYII: Cap-PF1.1) selected. Thus, Cap-PF1.1 was used as the seed for the next round of directed evolution. In each round, once a position yielded a beneficial substitution, it was fixed and excluded from further randomization. This lock-in strategy ensured that all seven positions were systematically optimized over seven rounds. At each round, the APPRAISE binding score for the lead peptide exceeded that of the previous round, yielding stepwise gains in predicted Ly6e binding (Figures 1D and 1E). Across the Cap-PF1.0→PF1.7 trajectory, the seven-residue insert evolved from RLPAYEI to WMDQIIY, showing alternating shifts in hydropathy (grand average of hydropathy [GRAVY])^45^ and net charge (Figure S2). The initial two rounds increased hydrophobicity by replacing a negatively charged residue (E→I) and a hydrophobic residue with a bulkier hydrophobic amino acid (L→M), setting the net charge to +1. A polar substitution (A→Q) then reduced the GRAVY value without affecting the charge, followed by Y→I, which restored hydrophobicity. Introduction of a negative charge (P→D) dropped the net charge from +1 to 0 and decreased the GRAVY value. The substitution I→Y further lowered hydrophobicity while leaving the charge unaltered. Finally, R→W shifted the net charge to −1 while partially restoring hydrophobicity. Thus, overall, the net charge progressed 0 → +1 → 0 → −1, and hydrophobicity oscillated accordingly, revealing a compensate-and-balance pattern that converged on Cap-PF1.7 (WMDQIIY).

The final evolved variant peptide WMDQIIY was named Cap-PF1.7. To validate the interaction between Ly6e and Cap-PF1.7, *in vitro* potency assays^31^ were performed. HEK293T cells were transiently transfected with plasmids encoding the Syrian hamster *Ly6e* and the effects on binding and transduction by Cap-PF1.7 capsids evaluated. Because *Ly6e* has a human ortholog (*LY6E*), we generated LY6E-knockout HEK293T cells to prevent cross-reactivity. Under these conditions, Cap-PF1.7 showed markedly increased binding and transduction in a Ly6e-dependent manner (Figures 1F and 1G), supporting a role for Ly6e in Cap-PF1.7-mediated entry.

### The evolved AAV capsid variant crosses the BBB of the Syrian hamster

**Figure 2:**
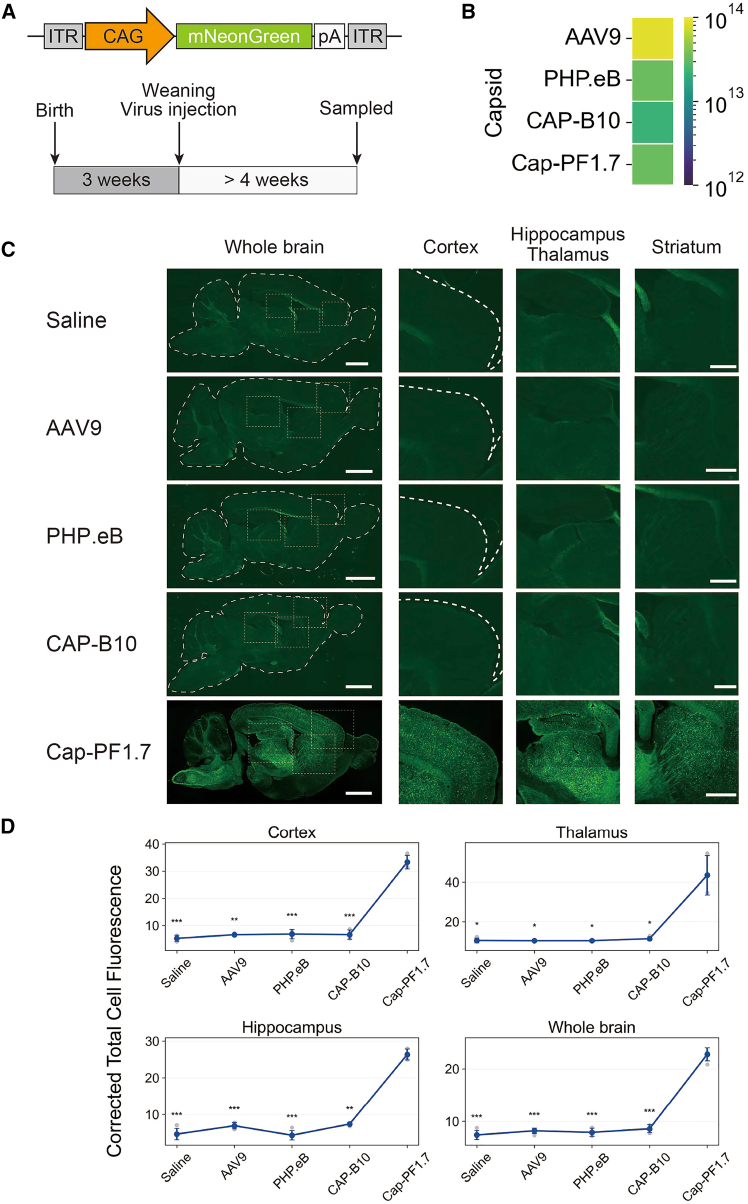
AAV.Cap-PF1.7 crosses the blood-brain barrier (BBB) of the Syrian hamster (A) Experimental scheme to assess capsid tropism for the central nervous system (CNS) *in vivo*. The AAV genome was engineered to express mNeonGreen under the control of a CAG promoter. Vectors were administered systemically to weaning Syrian hamsters via the retro-orbital sinus. Animals were sampled ≥4 weeks post-injection, and mNeonGreen expression in the CNS was examined by fluorescence microscopy. (B) Evaluation of the impact of peptide insertion on capsid fitness based on production yield (v.g./mL/20 cm dish). The color bar indicates the mean vector yield from 2 to 4 independent preparations per variant. (C) Fluorescence imaging of Syrian hamster brains. AAV9, AAV-PHP.eB, AAV.CAP-B10, and Cap-PF1.7 packaging CAG–mNeonGreen were administered intravenously at 1 × 10^13^ v.g. per animal (*n* = 3 per condition). Scale bars: 3 mm for whole brain images and 1 mm for higher-magnification images. (D) Fluorescence intensity was quantified in the indicated brain regions (cortex, thalamus, hippocampus, and whole brain) following administration of AAV vectors packaged with the indicated capsids. Each dot represents an individual animal; bars represent mean ± SD. Asterisks indicate comparisons of Cap-PF1.7 versus each of the other capsids within each brain region (∗*p* < 0.05, ∗∗*p* < 0.01; Welch’s two-sided *t* test).

To evaluate *in vivo* performance, we assessed the tissue tropism of Cap-PF1.7 after systemic administration in Syrian hamsters. For comparison with capsids developed for CNS tropism in mice, AAV9, AAV-PHP.eB, AAV.CAP-B10, and Cap-PF1.7 were used to package a reporter in which mNeonGreen was expressed under the ubiquitous CAG promoter^41^ (Figure 2A). All capsids produced virus with comparable efficiencies (Figure 2B), indicating that Cap-PF1.7 did not impair AAV capsid fitness. In each case, 1 × 10^13^ vector genomes (v.g.) of AAV variants were intravenously administered to 3-week-old hamsters. The hamsters were weaned and large enough to permit retro-orbital injection into the retro-orbital sinus (behind the globe of the eye). Transduction efficiency was assessed 4 weeks post-injection by monitoring mNeonGreen expression. As a result, AAV9, AAV-PHP.eB, and AAV.CAP-B10 failed to transduce the Syrian hamster brain, whereas Cap-PF1.7 efficiently transduced the adult CNS, with broad mNeonGreen expression in the cortex, hippocampus, and thalamus (Figures 2C and 2D). Moreover, strong tropism for fiber tracts, such as the striatum and the dorsal part of the spinal cord (spinal trigeminal nucleus), was also observed (Figure S3). To determine the cell types transduced by AAV.Cap-PF1.7, we performed Nissl staining in brain regions that exhibited strong transduction. Quantitative analysis revealed that mNeonGreen expression colocalized with Nissl-positive neurons in 91.6% of cells in the hippocampus, 70.7% in the cortex, and 93.3% in the thalamus (Figure S4A). These results indicate that neurons are the principal cellular targets of AAV.Cap-PF1.7 in the hamster brain. To assess potential off-target infection, we examined mNeonGreen expression in peripheral tissues and found that AAV.Cap-PF1.7 showed limited off-target infection in the liver (Figure S4B).

These results show that AAV.Cap-PF1.7 is an engineered capsid capable of crossing the BBB in Syrian hamsters and mediating widespread neuronal transduction throughout the CNS.

### Changes in tropism throughout the evolution process

**Figure 3:**
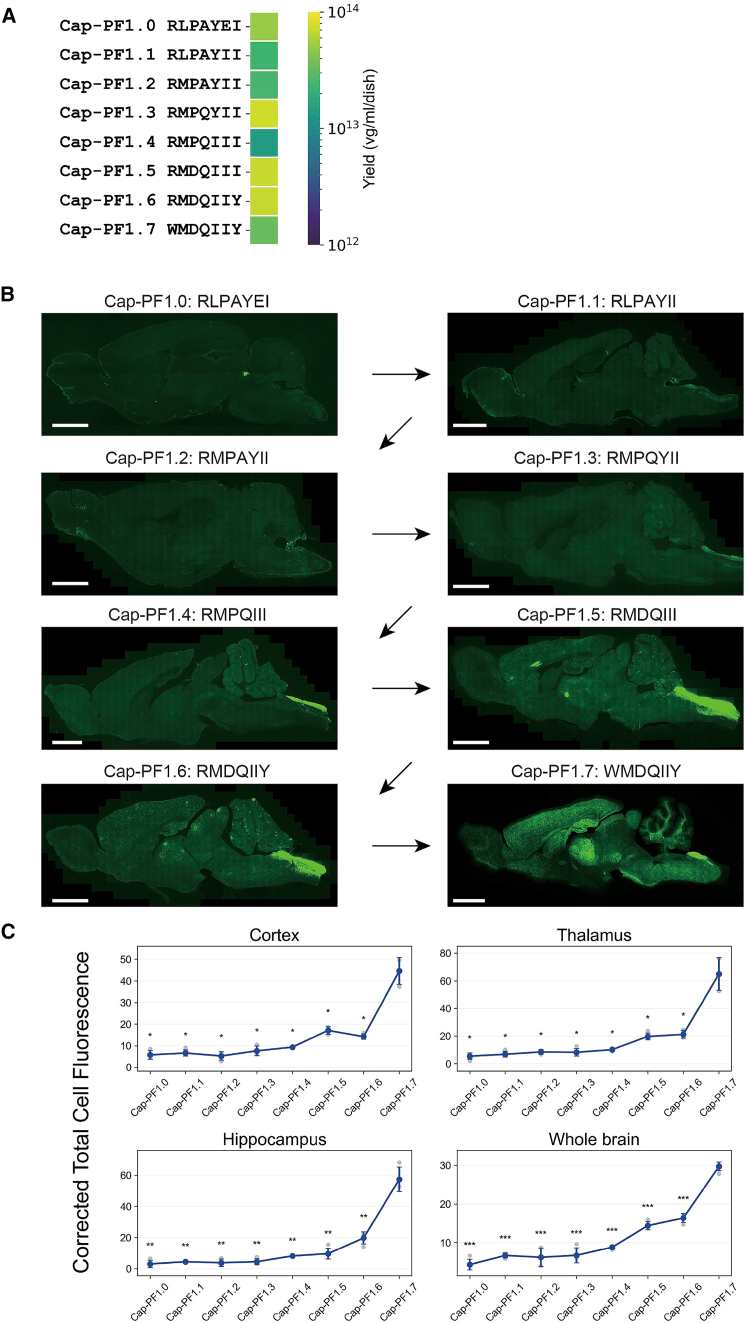
Directed evolution enhances the infectivity of AAV.Cap-PF1.7 within the central nervous system (CNS) (A) Evaluation of the impact of peptide insertion on capsid fitness based on production yield (v.g./mL/20 cm dish). The color bar indicates the mean vector yield from 2 to 4 independent preparations per variant. (B) Representative peptide-binder capsids from each round of directed evolution were used to package mNeonGreen under the control of the ubiquitous CAG promoter. Vector genomes were administered intravenously to Syrian hamsters at a dose of 1 × 10^13^ v.g. per animal (*n* = 3 per condition). Four weeks after administration, transgene expression was assessed by visualizing mNeonGreen fluorescence throughout the brain, demonstrating that the directed evolution process increased potency. Scale bars, 3 mm. (C) Corrected total cell fluorescence was quantified in the indicated brain regions (cortex, thalamus, hippocampus, and whole brain) following administration of AAV vectors packaged with Cap-PF1 variants. Each dot represents an individual animal; bars represent mean ± SD. Asterisks indicate comparisons of Cap-PF1.7 versus each of the other capsids within each brain region (∗*p* < 0.05, ∗∗*p* < 0.01, ∗∗∗*p* < 0.001; Welch’s two-sided *t* test).

To examine how CNS tropism emerged across the Cap-PF1.x optimization trajectory, we packaged pAAV-CAG-mNeonGreen into capsids displaying each intermediate peptide and evaluated CNS transduction under the same conditions as in Figure 2A. Across the series (Cap-PF1.0 to Cap-PF1.7), all variants yielded comparable vector production, indicating that the mutations introduced during evolution did not compromise capsid fitness (Figure 3A).

Cap-PF1.0, the top-ranked APPRAISE peptide from an initial pool of 100 random peptides, displayed no detectable mNeonGreen signal in the CNS. Similarly, Cap-PF1.1 and Cap-PF1.2 showed no measurable transduction. A faint but spatially restricted signal in the dorsal spinal cord was evident for Cap-PF1.3, which was more intense for Cap-PF1.4. Cap-PF1.5 showed robust parenchymal transduction in multiple brain regions, including cortex, thalamus and hippocampus, which was further intensified for Cap-PF1.6. The transition from Cap-PF1.6 to Cap-PF1.7 produced a marked, brain-wide increase in transduction, consistent with a late-stage consolidation of CNS tropism across regions (Figures 3B and 3C).

### Interaction of the VP3 trimer complex of Cap-PF1.7 with Ly6e

We next interrogated the structural basis of the Cap-PF1.7-Ly6e interaction in a two-step process. Firstly, the candidate binding mode for the Cap-PF1.7-Ly6e complex was generated and evaluated computationally. Secondly, experimental validation was performed using a cell-based potency assay.

**Figure 4:**
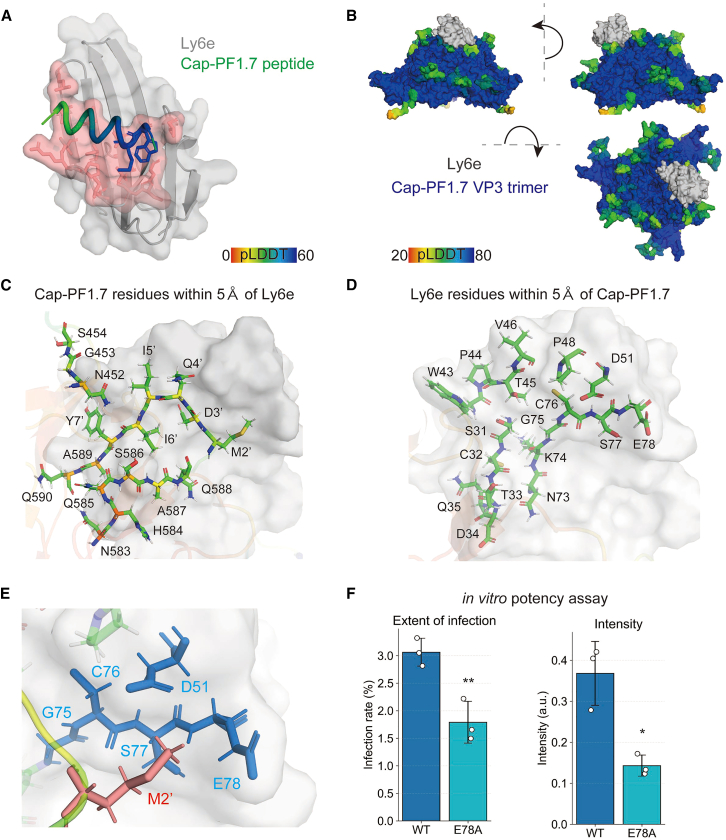
Structural analyses of the binding mode between AAV.Cap-PF1.7 and Ly6e (A) AlphaFold3-Multimer-predicted complex of Ly6e with the Cap-PF1.7 peptide. The peptide is color-coded by per-residue model confidence (i.e., pLDDT score). Ly6e residues containing ≥2 atoms within 5 Å of any peptide atom are highlighted in red and defined as interface residues. High-confidence residues Trp1′ and Met2′ of Cap-PF1.7 are shown as sticks. (B) Overall binding mode of the Cap-PF1.7-bearing VP3 trimer in complex with Ly6e. The AlphaFold3-Multimer prediction from (A) was integrated with the Cap-PF1.7 VP3 trimer model, and loop structures at the binding interface were refined using RosettaRemodel within the AAV trimeric context. The final model was further optimized using the Rosetta fast-relax protocol. (C) Close-up view of the Cap-PF1.7-Ly6e binding interface. Cap-PF1.7 residues containing ≥2 atoms within 5 Å of Ly6e are highlighted. (D) Close-up view of the Cap-PF1.7-Ly6e binding interface from the Ly6e side. Ly6e residues containing ≥2 atoms within 5 Å of Cap-PF1.7 are highlighted. (E) Interface detail showing Ly6e residues (blue) with ≥2 atoms within 5 Å of Met2′ of the Cap-PF1.7 peptide (red). The VP3 loop structure is shown in yellow. (F) Functional validation of the interface by Ly6e mutagenesis. Substitution of the top-contact residue (E78A) reduced potency in a cell-culture infectivity assay. Left, percentage of infected cells (*n* = 3). Right, total mNeonGreen signal intensity per positive area (*n* = 3). Data are presented as mean ± SD. Asterisks indicate statistical significance versus the indicated control (∗*p* < 0.05, ∗∗*p* < 0.01; Welch’s two-sided *t* test).

For the computational analysis, the Cap-PF1.7-Ly6e binding mode was analyzed with AlphaFold3-Multimer.^30^ In this model, the peptide fits into the globular extracellular domain of Ly6e (Figure 4A). Within Cap-PF1.7, Trp1′ and Met2′ were identified as high-confidence positions using the predicted local distance difference test (pLDDT) metric. To incorporate the receptor interaction into the native capsid context, the Cap-PF1.7 peptide and Ly6e were modeled on the AAV threefold-symmetry spike. Because the AAV VP3 trimer (∼200 kDa) is a large protein complex, AlphaFold3-multimer did not capture direct contacts between Ly6e and the VP3 trimer bearing Cap-PF1.7. Therefore an integrative structural modeling pipeline was employed similar to that described by Shay et al.^40^ Firstly, the AlphaFold-predicted AAV VP3 trimer was structurally aligned with the AlphaFold-predicted receptor-peptide complex through the high-confidence Trp1′ and Met2′ residues of Cap-PF1.7. Secondly, to refine the geometry of the inserted loop, the entire Cap-PF1.7 sequence was optimized using RosettaRemodel,^46^ with Ala587 and Ala589 (VP1 indices) serving as anchor residues. Finally, the resulting models were further refined using the Rosetta fast-relax protocol to minimize total energy and improve local structural accuracy (Figure 4B). To identify the interface between the VP3 trimer and Ly6e, we defined contacting residues as those containing at least two atoms within 5 Å of each other. The final model revealed 17 capsid residues at the Ly6e interface, including all amino acids of the Cap-PF1.7 peptide except Trp1’ (Figure 4C). Conversely, 17 Ly6e residues were found in contact with the VP3 trimer (Figure 4D). Based on this structural analysis, Met2′ of Cap-PF1.7 showed a high pLDDT score and multiple contacts with Ly6e residues. These findings suggested Met2′ plays an important role in generating the interacting surface. Among the five Ly6e residues contacting Met2′, Glu78 exhibited the highest number of atomic contacts (Figure 4E).

To test the functional relevance of this interface an E78A substitution was introduced in Ly6e and an *in vitro* potency assay performed. The mutation markedly reduced both the infection rate and mNeonGreen signal intensity compared with wild-type Ly6e, supporting the predicted binding mode and implicating Glu78 as a key determinant of the Cap-PF1.7-Ly6e interaction (Figure 4F).

### An AAV capsid variant that interacts with human LY6E

**Figure 5:**
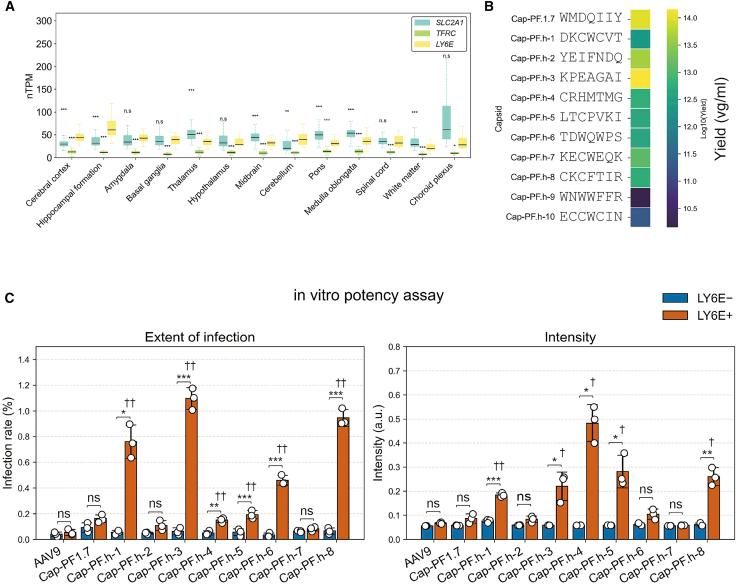
AAV capsid variant that interacts with human LY6E (A) Comparison of *SLC2A1*, *TFRC*, and *LY6E* mRNA expression levels across human brain regions using RNA expression data from the human protein atlas (https://www.proteinatlas.org). *SLC2A1* and *TFRC* are representative marker genes of the blood-brain barrier (BBB). Asterisks indicate comparisons between *LY6E* and each of the other genes within the same brain region (∗*p* < 0.05, ∗∗*p* < 0.01, ∗∗∗*p* < 0.001; Mann-Whitney U test with Benjamini-Hochberg correction). (B) Impact of peptide insertion on capsid fitness, assessed by production yield (v.g./mL/20 cm dish). The color bar indicates the mean vector yield from 2 to 4 independent preparations per variant. (C) Bright-field and mNeonGreen images were quantified to calculate extent of infection (infection rate, %; left) and intensity (brightness per transduced area, a.u.; right) for AAV9 and Cap-PF.h variants under LY6E-KO (LY6E−) or LY6E-expressing (LY6E+) conditions. Bars represent mean ± SD; open circles denote individual image measurements. Asterisks indicate comparisons between LY6E− and LY6E+ within the same capsid (*p* < 0.05 (∗), *p* < 0.01 (∗∗), and *p* < 0.001 (∗∗∗)). Daggers (†) indicate comparisons of each capsid under LY6E + conditions versus AAV9 (LY6E+) (ns, not significant*; p* < 0.05 (†), *p* < 0.01 (††); Welch’s two-sided *t* test with Holm-Bonferroni correction).

Finally, we explored whether receptor-dependent transduction could extend to the human Ly6 family member *LY6E*. To assess *LY6E* expression in the human brain, the human protein atlas^47^^,^^48^^,^^49^ was consulted. *LY6E* mRNA levels across brain regions were compared with BBB marker genes *TFRC* and *SLC2A1*. *LY6E* was found to be expressed at higher levels than *TFRC* in all regions and at levels comparable to or exceeding *SLC2A1* in many regions (Figure 5A). Importantly, *LY6E* expression has also been reported in human brain vascular endothelial cells in a cerebrovascular single-nucleus transcriptomic atlas,^50^ supporting its relevance as a BBB-facing target. These findings motivated us to test whether LY6E-binding capsids can enhance transduction in an LY6E-dependent *in vitro* setting. Ten candidate LY6E-binding variants were generated using EvoPRAISE. Using the same workflow as for Syrian hamster Ly6e (Figure 1), we performed iterative *in silico* evolution starting from multiple randomly generated 7-mer seed peptides against the human LY6E extracellular domain (Figure S5). Cap-PF.h-9 and Cap-PF.h-10 were excluded from further assays due to low AAV yields, consistent with impaired capsid fitness and/or structural destabilization (Figure 5B). *In vitro* potency assays on the remaining eight variants were performed under LY6E-KO (LY6E−) or LY6E-expressing (LY6E+) conditions. Under these settings, Cap-PF.h-1, Cap-PF.h-3, Cap-PF.h-4, Cap-PF.h-5, and Cap-PF.h-8 showed significantly higher extent of infection and signal intensity than AAV9 (Figure 5C). Together, these results support LY6E-dependent enhancement of transduction for multiple variants *in vitro*. Whether LY6E-targeting capsids can mediate BBB delivery *in vivo*, and the extent to which this approach transfers to other receptors, will require separate validation.

## Discussion

Crossing the BBB to deliver genes into the CNS enables mechanistic studies of brain physiology and supports therapeutic development. In hibernation biology, mechanistic genetic approaches have been particularly difficult to implement because many hibernating species are non-model organisms that lack readily adaptable genetic toolkits. Consequently, it has been challenging to directly test causal relationships between candidate genes and hibernation phenotypes. It has also been difficult to visualize and manipulate specific cell populations implicated in the induction, maintenance, and recovery phases. By enabling non-invasive, brain-wide gene delivery in a hibernator-relevant species, Cap-PF1.7 helps overcome these technical barriers and supports a causality-driven experimental framework for hibernation research. Systemic CNS delivery would allow direct causal testing of candidate genes involved in hibernation-related states. Cap-PF1.7 could also package AAV-based tools that label active neurons,^51^^,^^52^ which would make it possible to visualize neuronal populations correlated with induction, maintenance, and arousal or recovery. In addition, combining Cap-PF1.7 with cell-type-specific promoters or enhancers and optical manipulation technologies could enable researchers to turn defined neuronal populations on or off during hibernation.^53^^,^^54^ This would permit direct tests of causal links between particular cell types and hibernation phenotypes. Many research groups have modified AAV capsids to enhance CNS tropism.^10^^,^^11^^,^^12^^,^^55^^,^^56^^,^^57^^,^^58^ In mice, large-scale *in vivo* library screens can identify BBB-penetrant variants, such as AAV-PHP.eB in the C57BL/6 strain. However, translating these findings to non-model species, including humans, remains challenging due to limited cohort sizes and the lack of strain-matched genetic tools. Mechanism-guided capsid engineering has recently advanced through approaches that explicitly design capsid-receptor interactions to enable BBB crossing, providing vectors with defined entry mechanisms and predictable tropisms.^31^^,^^32^ These studies further highlight that several prominent mouse-derived BBB-crossing capsids depend on LY6A or LY6C1, which are absent in primates, helping to explain limitations in translation across species. In our Ly6e-focused study, EvoPRAISE served as a practical complement to large-scale *in vivo* library screening by prioritizing a small number of species-matched candidates when material was limiting and repeated rounds of selection were not readily feasible. We also note that oligo-pool random peptide libraries constructed with degenerate codons such as NNK or NNS can introduce premature stop codons and reduce the effective yield of full-length randomized peptides as library length increases, whereas our *in silico* design operates directly on amino acid sequences and avoids stop-codon constraints at the design stage. In this Ly6e-focused study, a receptor-guided design strategy allowed us to prioritize candidates *in silico* rather than testing large libraries in animals. Using this approach, limited *in vivo* resources were focused on a small number of species-matched sequences for the intended model and target receptor.

Recent computational methods for protein binders use structure prediction,^59^ diffusion models,^33^^,^^35^^,^^60^ language models,^34^^,^^61^ and physics-based modeling^62^ to propose sequences with high predicted affinity. However, high affinity alone is often insufficient in physiological contexts, particularly near membranes, where receptor topology, membrane curvature, and the glycocalyx create steric barriers that hinder productive binding. In this study, we used APPRAISE to prioritize peptide-receptor pairs by combining AlphaFold-based complex modeling with rapid analyses of surface complementarity and steric accessibility, thereby ranking candidates in a capsid-display context.

Because APPRAISE relies on competitive pairwise modeling within a peptide pool, its ranking performance depends on the composition of the candidate set. In our Ly6e-focused setting, APPRAISE was most informative once a promising candidate sequence had been identified, whereas fully *de novo* discovery from small, uninformative pools was less reliable. To iteratively refine candidates in this study, we coupled APPRAISE with an *in silico* mutagenesis-and-selection loop (EvoPRAISE). In each round, sequence diversity was introduced around the current lead (e.g., single-site saturation mutagenesis at selected positions), followed by APPRAISE-based competitive modeling to prioritize improved variants. Repeating this process enabled stepwise refinement of Ly6e-interacting peptide inserts while limiting the number of variants carried forward to experimental testing.

In this study, we identify Cap-PF1.7, an evolved AAV9-derived capsid variant that enables widespread CNS transduction in Syrian hamsters following intravenous administration. Within the CNS, we observed regionally enriched tropism in the cortex, hippocampus, thalamus, striatum, and rostral dorsal spinal cord. These regional differences are plausibly attributable to spatial heterogeneity in endothelial receptor expression, including Ly6e, rather than extensive post-entry diffusion. Consistent with this interpretation, post-entry diffusion has been reported to be restricted by electrostatic interactions with extracellular matrix components, including perineuronal nets and heparan sulfate proteoglycans.^63^ Along the evolutionary trajectory toward Cap-PF1.7, the peptide also underwent biochemical adaptation. Initial increases in hydrophobicity promoted interface formation and stabilization, while subsequent reintroduction of polarity restored solubility and conformational flexibility. A recurring pattern emerged, characterized by a hydrophobic core surrounded by a polar rim, which is typical of protein-protein interfaces. Oscillations in GRAVY values are consistent with exploratory adaptation and suggest stepwise testing of local compatibility with the receptor and surrounding structures before convergence on a functional optimum.^64^^,^^65^ In this trajectory, the combination of saturation mutagenesis with iterative selection likely contributed to diversification and helped mitigate premature convergence on local optima in the APPRAISE scoring landscape.

### Limitations of the study

Despite the success of the Ly6e-focused study, practical constraints remain. Firstly, the performance of peptides obtained from a random input library can depend on library size. Very small libraries increase the risk of trapping at local optima on the binding score landscape. Because in our implementation, the search sampled only a small fraction of the theoretical 20^*L*^ combinatorial space (where *L* is the peptide length in amino acids), top candidates may converge to distinct local optima and therefore appear highly sequence-divergent (Figure 5B). Increasing the size and diversity of the initial seed pool, or repeating the pipeline with multiple independent seed sets, should broaden the explored sequence space and improve the likelihood of identifying a suitable lead. Consistent with this, in our LY6E ortholog test, roughly half of the EvoPRAISE-designed candidates outperformed AAV9 (Figure 5C), supporting the practicality of using multiple independent seed sets to increase the likelihood of identifying a viable starting sequence. These considerations imply that the peptides obtained are unlikely to represent globally optimal solutions. Accordingly, our goal here was not to exhaustively enumerate the full combinatorial space. Rather, it is designed to generate a tractable set of candidate scaffolds *in silico* that can be refined with limited downstream screening. The second issue with EvoPRAISE is that runtime is dominated by AlphaFold Multimer inference. Pairwise competition scales quadratically with the number of variants, which is impractical for very large libraries. For single site saturation per round, the modeled variants per round scale approximately linearly with peptide length, about 19 times *L* for one site scans. To address the computational cost of EvoPRAISE and to enable integration with experimental workflows, several complementary strategies can be applied. More efficient evolutionary search algorithms can be used to reduce computational expense, such as simple genetic algorithms with small populations, low mutation rates, and early stopping criteria to prevent unnecessary modeling. In addition, a compact candidate peptide binder library generated by EvoPRAISE can be screened *in vivo* using a focused design strategy. This approach is more practical for non-model species and can provide quantitative feedback for subsequent computational iterations. Finally, EvoPRAISE inherits uncertainties from predictive models and may not account for downstream barriers such as endocytosis, endosomal escape, intracellular trafficking, and uncoating. In addition, target selection itself remains a major source of uncertainty. The development of reliable prediction methods and databases for identifying proteins suitable as AAV targets is highly desirable. Resources such as the Cell Surface Protein Atlas,^66^ and brain vascular proteomic atlases^67^ provide a foundation for identifying potential AAV receptors. These datasets allow candidate proteins to be filtered by surface localization and endothelial enrichment, improving the selection of receptors that are accessible at the BBB for experimental validation. Accordingly, the likelihood that a receptor-targeted design yields the intended tropism is expected to vary substantially with the target receptor and its expression pattern across relevant cell types and tissues. It will therefore be important to evaluate performance in more physiologically relevant systems that better approximate *in vivo* conditions, including iPSC-derived brain microvascular endothelial cells, BBB-on-chip platforms, vascularized brain organoids, and non-neural organoids to assess potential off-target transduction.^68^^,^^69^^,^^70^^,^^71^

In summary, EvoPRAISE couples competitive structure modeling with iterative *in silico*-guided refinement to prioritize peptide inserts for capsid display. In this Ly6e/LY6E-focused study, this workflow enabled stepwise optimization from initial peptide candidates to variants that support receptor-dependent transduction and enhanced CNS delivery in Syrian hamsters, while also identifying multiple LY6E-dependent variants *in vitro*. While the computational workflow is reusable, the experimental validation presented here is focused on Ly6e/LY6E. Establishing broader generality across unrelated receptors will require prospective applications to additional targets using matched receptor-negative/receptor-positive assays.

## Resource availability

### Lead contact

Further information and requests for resources and reagents should be directed to and fulfilled by the lead contact, Hiroaki Ono (hiroaki.ono.pf@gmail.com).

### Materials availability

Plasmids generated in this study have been deposited to Addgene where indicated in the key resources table. Additional plasmids and AAV reagents generated in this study will be made available by the lead contact upon reasonable request.

### Data and code availability

• Peptide sequence data generated in this study are provided in the figures. Publicly available datasets used in this study include UniProt protein sequence records for mouse Ly6A (UniProt: P05533), Syrian hamster Ly6e (UniProt: A0A1U7Q6V5) and human LY6E (UniProt: Q16553), as well as human brain expression data from the Human Protein Atlas (genes analyzed: LY6E, TFRC, and SLC2A1; https://www.proteinatlas.org/humanproteome/brain). Structural model files generated in this study and image data underlying the figures are available from the lead contact upon reasonable request.
• Custom Python code used for analysis has been deposited in Mendeley Data and is publicly available as of the date of publication at https://doi.org/10.17632/g49ycssrnv.1.
• Any additional information required to reanalyze the data reported in this paper is available from the lead contact upon request. Plasmids generated in this study are available from Addgene as listed in the key resources table.

## Acknowledgments

This work was supported by the 10.13039/501100001691Japan Society for the Promotion of Science (10.13039/501100001691JSPS) Grant-in-Aid for Early-Career Scientists (23K14130) (to H.O.), RIKEN GAP Fund (to H.O.), the JSPS Grant-in-Aid for Transformative Research Areas (A) (23H04941) (to G.A.S.), and the Suntory Rising Stars Encouragement Program in Life Sciences (to G.A.S.).

## Author contributions

H.O. designed the study. H.O. performed the animal experiments. H.O. and S.F. performed the histology analysis. H.O. and S.F. generated AAVs. H.O. performed the computational peptide design. H.O. wrote the original manuscript, which was reviewed and edited by G.A.S. G.A.S. supervised the study.

## Declaration of interests

H.O. and G.A.S. are inventors on a patent application related to the technology described in this manuscript.

## Declaration of generative AI and AI-assisted technologies in the writing process

During the preparation of this work, the authors used ChatGPT in order to improve grammar and writing clarity. After using this tool/service, the authors reviewed and edited the content as needed and take full responsibility for the content of the publication.

## STAR★Methods

### Key resources table

**Table:** 

REAGENT or RESOURCE	SOURCE	IDENTIFIER
**Bacterial and viral strains**		

AAV.Cap-PF1.0-CAG-mNeonGreen	This paper	N/A
AAV.Cap-PF1.1-CAG-mNeonGreen	This paper	N/A
AAV.Cap-PF1.2-CAG-mNeonGreen	This paper	N/A
AAV.Cap-PF1.3-CAG-mNeonGreen	This paper	N/A
AAV.Cap-PF1.4-CAG-mNeonGreen	This paper	N/A
AAV.Cap-PF1.5-CAG-mNeonGreen	This paper	N/A
AAV.Cap-PF1.6-CAG-mNeonGreen	This paper	N/A
AAV.Cap-PF1.7-CAG-mNeonGreen	This paper	N/A
AAV.Cap-PF.h-1-CAG-mNeonGreen	This paper	N/A
AAV.Cap-PF.h-2-CAG-mNeonGreen	This paper	N/A
AAV.Cap-PF.h-3-CAG-mNeonGreen	This paper	N/A
AAV.Cap-PF.h-4-CAG-mNeonGreen	This paper	N/A
AAV.Cap-PF.h-5-CAG-mNeonGreen	This paper	N/A
AAV.Cap-PF.h-6-CAG-mNeonGreen	This paper	N/A
AAV.Cap-PF.h-7-CAG-mNeonGreen	This paper	N/A
AAV.Cap-PF.h-8-CAG-mNeonGreen	This paper	N/A
AAV.Cap-PF.h-9-CAG-mNeonGreen	This paper	N/A
AAV.Cap-PF.h-10-CAG-mNeonGreen	This paper	N/A
AAV9-CAG-mNeonGreen	This paper	N/A
AAV-PHP.eB-CAG-mNeonGreen	This paper	N/A
AAV.CAP-B10-CAG-mNeonGreen	This paper	N/A
LV-U6-gRNA(hLy6e_grna1)-Cas9-mCherry	This paper	N/A
LV-U6-gRNA(hLy6e_grna2)-Cas9-mCherry	This paper	N/A
LV-U6-gRNA(hLy6e_grna3)-Cas9-mCherry	This paper	N/A

**Biological samples**		

pUCmini-iCAP-PHP.eB	Chan et al.^41^	Addgene Plasmid #103005
pAAV-CAG-mNeonGreen	Chan et al.^41^	Addgene Plasmid #99134
pUCmini-iCAP-AAV.CAP-B10	Goertsen et al.^12^	Addgene Plasmid #175004
pAAV2/9n	Unpublished	Addgene Plasmid #112865
pCAG-HIVgp	Miyoshi et al.^72^	RIKEN DNA bank RDB04394
pCMV-SVF-P2A-VSV-G-pRSV-REV	Miyoshi et al.^72^	RIKEN DNA bank RDB04393
AAV:ITR-U6-sgRNA(backbone)-pCBh-Cre-WPRE-hGHpA-ITR	Platt et al.^73^	Addgene Plasmid #60229
NEB® Stable Competent E. coli (High Efficiency)	New England Biolabs	Cat# C3040H
pCap-PF1.0	This paper	N/A
pCap-PF1.1	This paper	N/A
pCap-PF1.2	This paper	N/A
pCap-PF1.3	This paper	N/A
pCap-PF1.4	This paper	N/A
pCap-PF1.5	This paper	N/A
pCap-PF1.6	This paper	N/A
pCap-PF1.7	This paper	Addgene Plasmid #252191
pCap-PF.h-1	This paper	Addgene Plasmid #252192
pCap-PF.h-2	This paper	N/A
pCap-PF.h-3	This paper	Addgene Plasmid #252193
pCap-PF.h-4	This paper	Addgene Plasmid #252194
pCap-PF.h-5	This paper	Addgene Plasmid #252195
pCap-PF.h-6	This paper	N/A
pCap-PF.h-7	This paper	N/A
pCap-PF.h-8	This paper	Addgene Plasmid #252196
pCap-PF.h-9	This paper	N/A
pCap-PF.h-10	This paper	N/A

**Chemicals, peptides, and recombinant proteins**		

DMEM	Gibco	Cat# 11995-065
DMEM, high glucose, GlutaMAX Supplement	Gibco	Cat# 10566016
FluoroBrite™ DMEM	Gibco	Cat# A1896701
NeuroTrace™ 530/615 Red Fluorescent Nissl Stain - Solution in DMSO	Invitrogen	Cat# N21482
FBS	Biosera	Cat# 556-33865
Trypsin-EDTA (0.25%), phenol red	Gibco	Cat# 25200056
MEM Non-essential Amino Acids Solution (×100)	FUJIFILM Wako Pure Chemical Corporation	Cat# 139-15651
OptiPrep	Cosmo Bio	Cat# 1893
Proteinase K	Tokyo Chemical Industry Co.	Cat# P3013
Benzonase	Sigma-Aldrich	Cat# E8263-5KU
TB Green® Premix Ex Taq™ GC (Perfect Real Time)	TaKaRa Bio Inc	Cat# RR071A
PrimeSTAR® Max DNA Polymerase	TaKaRa Bio Inc	Cat# R045A
DNA Ligation Kit	TaKaRa Bio Inc	Cat# 6023
T4 Polynucleotide Kinase	TaKaRa Bio Inc	Cat# 2021S
100bp DNA Ladder PLUS	NIPPON Genetics Co, Ltd.	Cat# NE-MWD100P
PEI MAX	Cosmo Bio	Cat# 24765-100
Lipofectamine 3000 Reagent	Invitrogen	Cat# L3000015

**Critical commercial assays**		

Wizard Plus SV Minipreps DNA Purification Systems	Promega	Cat# A1460
AAVpro Helper Free System (AAV9)	TaKaRa Bio Inc	Cat# 6690
PureLink HiPure Plasmid Midiprep Kit	Invitrogen	Cat# K210004
QIAamp DNA Mini Kit	QIAGEN	Cat# 56304
RNeasy Micro Kit	QIAGEN	Cat# 74104
QuantiTect Reverse Transcription Kit	QIAGEN	Cat# 205310

**Oligonucleotides**		

Sequences of primers; see Table S1	This paper	N/A

**Experimental models: Cell lines**		

HEK293T	RIKEN BioResource Research Center	RCB2202
HEK293T LY6E-KO	This paper	N/A
AAVpro 293T	TaKaRa Bio Inc	Cat# 632273

**Experimental models: Organisms/strains**		

Syrian hamster	Japan SLC, Inc.	Slc:Syrian
C57BL/6J	Oriental Yeast Co., Ltd.	N/A

**Software and algorithms**		

ImageJ	Schneider et al.	https://imagej.nih.gov/ij/
Python 3.9.12	Van Rossum, 1995	https://www.python.org/
Numpy 1.26.3	Harris et al., 2020	https://github.com/numpy/numpy
Pandas 2.1.4	McKinney, 2010	https://github.com/pandas-dev/pandas
Matplotlib 3.8.0	Hunter, 2007	https://github.com/matplotlib/matplotlib

### Experimental model and study participant details

#### Animals

All Syrian hamsters were purchased from Japan SLC, Inc. as pregnant females. Animals were provided food and water *ad libitum* and housed under controlled environmental conditions, with an ambient temperature of 21°C, relative humidity of 50%, and a 12 h light and 12 h dark cycle (lights on at 8:00 a.m. and off at 8:00 p.m.). Pups were weaned at 3 weeks of age. All AAV injections used for experiments shown in Figures 1, 2, and 3 were performed immediately after weaning.

All animal experiments were approved by the Institutional Animal Care and Use Committee of the RIKEN Kobe Branch (approval no. A2022-08-17) and were conducted in accordance with the RIKEN Regulations for Animal Experiments.

### Method details

#### Evolutionary optimization combined with APPRAISE (EvoPRAISE) for AAVs

EvoPRAISE in this study comprised two processes: the identification of seed peptides and the evolution of these seed peptides. Custom Python code was used for the seed peptide identification process. In all, 100 variant peptides were generated by inserting a randomized sequence (7-mer) between AA588-589 (VP1 indices) of the AAV9 capsid. The APPRAISE method was employed to identify peptides that would serve as the seeds for directed evolution. Specifically, peptides with stronger affinity for the target sequence than AA587-594 of the AAV9 capsid (AQAQAQTG) and AA587-601 of PHP.eB (DGTLAVPFKAQAQTG) were identified. Affinity was evaluated using Delta_B as the metric, which is the default setting of the APPRAISE method. The chosen target sequences were the extracellular domains of hamster Ly6e (A0A1U7Q6V5_MESAU) (AA27-118) and human LY6E (Q16553 · LY6E_HUMAN) (AA21-112). The extracellular domains were determined as homologous sequences based on alignment with the AA27-110 peptide of mouse Ly6A (P05533 · LY6A_MOUSE), which was used in the demonstration of the APPRAISE method.

**Table:** 

Hamster Ly6e (A0A1U7Q6V5_MESAU) extracellular domain sequence
LVCFSCTDQKSNLKCLWPTVCPSSDNYCVTVSATAGFGDINLGYTLNKGCSEICPRENINVNLGVASVNSYCCRHSFCNISTAGLGLRASVP
Human LY6E (Q16553 · LY6E_HUMAN) extracellular domain sequence
LMCFSCLNQKSNLYCLKPTICSDQDNYCVTVSASAGIGNLVTFGHSLSKTCSPACPIPEGVNVGVASMGISCCQSFLCNFSAADGGLRASVT

The publicly available version of APPRAISE was used (available at https://github.com/GradinaruLab/APPRAISE). Briefly, FASTA-format files containing the amino acid sequence of the target receptor and peptide sequences were prepared for structural prediction. Batch-mode ColabFold (version 2.1.14) was used for complex modeling on Google Colaboratory equipped with an NVIDIA Tesla V100 SXM2 16 GB GPU or NVIDIA A100 Tensor Core 40 GB GPU. Unless otherwise specified, the AlphaFold model version alphafold-multimer-v2 was used as the default setting. Each model was recycled 3 times, and 5 models were generated per competition. Quantitative analyses of the resulting structures were performed in PyMOL (version 2.3.3) using custom scripts originally developed by Ding et al.^39^ The following parameters were calculated: the number of peptide atoms at the receptor interface (*N* POI contact; defined by an interatomic distance cutoff of 5 Å); the number of peptide atoms clashing with the receptor (*N* POI clash; defined by a distance cutoff of 1 Å); the peptide binding angle (*θ*; defined as the angle between the vector from the receptor centroid to the anchor and the vector from the receptor centroid to the peptide centroid); and the peptide binding depth (*d*; defined as the difference between the distance from the receptor center to the closest peptide atom and the minor radius of the receptor ellipsoid hull, normalized by the minor radius). The minor radius of the receptor ellipsoid hull was calculated using HullRad 8.1, which yielded a value of 15.1 Å for Ly6e. Finally, the binding propensity metric *ΔB*^POI,competitor^ was computed as the difference in total binding scores between competing models. In the first round, the ranking was generated by comparing each peptide against the PHP.eB peptide as the reference competitor.

To perform peptide evolution, a site-saturation mutagenesis library was generated using custom scripts. Each amino acid residue in the selected peptide was substituted with all possible amino acids at that position. The APPRAISE pipeline, using the same parameter settings as described above, was then applied to evaluate and rank the peptide variants based on their predicted affinity for the target proteins. For *Round N* (0 ≤ *N* ≤ *L*, where *L* is the peptide length in amino acids), each mutant library was compared against the top-ranked peptide from *Round N - 1* to identify improved variants. In *Round N + 1*, the mutated position identified in *Round N* was fixed, and site-saturation mutagenesis performed on the remaining *L−N* residues. Thus, the evolutionary process for the 7 amino acid peptide entailed 7 iterative rounds of mutation. The final peptide obtained represented the product of *in silico* directed evolution. All complex structures in this study were modeled using template-based modeling settings.

msa_mode = “MMseqs2 (UniRef+Environmental)”

num_models = 5

num_recycles = 20

stop_at_score = 100

use_custom_msa = False

use_amber = False

use_templates = True

model_type = “auto” or “alphafold2_multimer_v2”

#### Plasmid construction

All capsid variants were generated using a common mutagenesis strategy. First, the entire AAV9 capsid coding sequence was subcloned into the multiple cloning site of the pUC19 vector (pUC19-Cap9). The amino acid sequences of the peptide binders were converted into nucleotide sequences optimized for human codon usage. The optimized peptide-coding sequences were inserted into the peptide insertion site of pUC19-Cap9, located between residues 588 and 589 of the AAV9 capsid (VP1 indices), by vector PCR using PrimeStar Max DNA polymerase followed by blunt-end ligation. The mutated capsid fragments were amplified using primers flanking the mutagenized region and cloned into the corresponding site of the pUCmini-iCAP-PHP.eB (Addgene plasmid #103005). Sequences of all the oligonucleotide primers used for plasmid construction are given in Table S1.

All recombinant DNA experiments, including plasmid construction, were approved by the relevant institutional committee of the RIKEN Kobe Branch (approval no. K2022-004-13) and were conducted in accordance with institutional guidelines and regulations.

#### AAV preparation

The protocol for AAV production shown below was based on that described by Challis et al., 2019^74^ with modifications. AAVpro 293T was cultured in 150 mm dishes in a culture medium containing DMEM (high glucose) (Gibco), 10% (v/v) fetal bovine serum (FBS), and 1% penicillin-streptomycin (PS) at 37°C and 5% CO_2_. pAAV, capsid of interest, and pHelper plasmid were transfected into cells, reaching 90–100% confluency. A pAAV: capsid: pHelper plasmid ratio of 1:4:2 based on micrograms of DNA (i.e., 5.7 μg of pAAV, 22.8 μg of capsid, and 11.4 μg of pHelper) was used for the transfection. On the day following transfection, the culture medium was exchanged for a 20 mL culture medium containing DMEM (high glucose, GlutaMAX) (Gibco), 5% (v/v) FBS, 1% MEM Non-Essential Amino Acids solution (NEAA) (Wako), and 1% PS. After 72 h following transfection, the culture medium was collected and exchanged for 20 mL of fresh culture medium containing DMEM (high glucose, GlutaMAX), 5% (v/v) FBS, 1% MEM NEAA, and 1% PS. The collected culture medium was stored at 4°C. Cells were subsequently collected using a cell scraper 144 h after transfection. The collected culture medium obtained 72 h post-transfection was separated into supernatant and cell pellet by centrifugation (2000 x g, 20 min). Polyethylene glycol was added to the supernatant to a final concentration of 8%. The resulting solution was then incubated on ice for 2 h. The solution was centrifuged (5000 x g, 30 min) and the pellet suspended into 2 mL Tris MgCl_2_ (10 mM Tris pH 8.0, 2 mM MgCl_2_). The suspension was subjected to three rounds of freeze-thaw cycles. Benzonase (100 U/mL) was then added and the mixture incubated at 37°C for 1 h. The supernatant solution was mixed with the cell solution, and the resulting mixture centrifuged (2,000 x g, 10 min) to obtain the supernatant. Iodixanol density gradient solutions (15%, 25%, 40%, and 60% (wt/vol)) were prepared and poured into an Optiseal centrifuge tube (Beckman). Tubes were transferred to a Type 70 Ti rotor (Beckman) and centrifuged at 350,000 x g for 2 h 25 min. In each case the 40% iodixanol layer containing the virus was collected. The purified viral solution was obtained by ultrafiltration using an Amicon filter device (Merck) and resuspension into Dulbecco’s phosphate-buffered saline.

For AAV titration, purified virus solution was treated with Benzonase (0.05 U/mL, 37°C, 1 h) followed by Proteinase K (0.25 mg/mL, 37°C, 1 h). The viral genome was subsequently obtained by phenol-chloroform-isoamyl alcohol extraction followed by isopropanol precipitation. The titer (v.g./mL) of the AAV was calculated by quantifying the number of WPRE sequences in the sample using qPCR. pAAV-CAG-mNeonGreen (Addgene Plasmid #99134) was used as the standard. The qPCR protocol was as follows: 95°C for 60 s (initial denaturation) followed by 45 cycles of 95°C for 10 s, 60°C for 30 s using TB Green Premix Ex Taq GC (TaKaRa Bio Inc). The final purified virus samples were stored at −80°C. Titers of recombinant AAV vectors were determined by quantitative PCR.

#### AAV vector administration, tissue processing and imaging

In the hamster experiments, AAV vectors were administered intravenously via retro-orbital injection at doses of 1 × 10^13^ v.g. as indicated in the figures and corresponding legends. After 4 weeks of expression, hamsters and mice were anesthetized with isoflurane and transcardially perfused with approximately 50 mL of 10% sucrose and then another 50 mL of 4% paraformaldehyde (PFA) in 0.1 M phosphate-buffered saline pH 7.4 (PBS). Organs were post-fixed overnight in 4% PFA at 4°C, incubated overnight in 30% sucrose in 0.1 M PBS at 4°C. Finally, organs were cut into 50 μm sections using a Leica CM1950 Cryostat. Images were acquired with a fluorescence microscope (Axio Observer 7, ZEISS) and processed using ZEN 3.4 (Zeiss) and ImageJ software.

#### Lentivirus production

Lentivirus production was performed according to RIKEN’s protocol (https://dnaconda.riken.jp/Form_PDF/lntPrepen.pdf) with some modifications. HEK293T cells were seeded onto six 15 cm culture dishes (Falcon) at 1.69 × 10^7^ cells/dish. After 24 h incubation, the medium was changed to 19 mL Improved Minimum Essential Medium (IMEM) supplemented with 15 μM chloroquine (Wako) without PS and FBS. Cells were then incubated for an extra 1 h at 37°C, 5% CO_2_. Transfection was conducted using the calcium phosphate precipitation method. In brief, the plasmid mixture was prepared by mixing 281 μg target plasmid, 165 μg pCAG-HIVgp, and 165 μg pCMV-SVF-P2A-VSV-G-pRSV-REV, and made up to 7.5 mL with double distilled water. Next, 500 μL of 2.5 M CaCl_2_ (Wako) was applied to the bottom of the tube containing this plasmid mixture and vortexed vigorously for 10 s. An 8 mL aliquot of borate buffered saline (Wako) buffer was added dropwise to the plasmid mixture while gently mixing. After incubation at room temperature for 8 min, 2.7 mL of the plasmid mixture was applied to the HEK293T cells and the mixture incubated at 37°C, 5% CO_2_ for 1 h. 10% FBS was then added to medium. After incubating for 13 h, medium was changed to 15 mL IMEM containing 1.5 mM sodium butyrate (Wako) and 1.5 mM caffeine (Tokyo Chemical Industry). Following further incubation at 37°C, 5% CO_2_ for 34 h, the supernatants were collected and centrifuged (4°C, 900 g, 10 min) to remove cell debris. Finally, the supernatant was filtered through a 0.45 μm PES filter unit (Merck Millipore), divided into three 30 mL tubes (Beckman Coulter, 326823) by layering onto 10 mL of 10% sucrose solution, and centrifuged at 4°C, 15,000 rpm for 4 h in an ultracentrifuge (Beckman Coulter) to pellet the virus. The resulting pellet was vigorously resuspended in 3 mL of chilled PBS and 10 μL aliquots of the suspension were stored at −80°C.

Titer evaluation was performed to determine the infectious titer. HEK293T cells were seeded into 24-well plates (Corning) at 3.53 × 10^4^ cells/well. After 4 h of incubation, serially diluted virus solutions (0, 1,000, 2,000, 4,000, 8,000, 16,000, 32,000-fold dilution) were applied to the cells in a final volume of 0.5 mL per well, followed by incubation at 37°C in 5% CO_2_ for an additional 72 h. Cells were then detached with 0.25% trypsin-EDTA (Wako) and analyzed using Cell Sorter MA900 to quantify the percentage of fluorescent protein-positive cells. The infectious titer unit (IFU/mL) was calculated using the following formula:

$$\text{IFU}/\text{mL}=\frac{\left(\frac{20}{100}\right)\times 35000\;\text{cells}\times \text{estimated}\;\text{fold}\;\text{dilution}\;\left(\text{at}\;20\%\right)}{0.5\;\text{mL}}$$

where 0.5 mL corresponds to the volume of virus solution applied per well. The estimated fold dilution at 20% was determined by approximate linearization (Microsoft Excel) based on infection efficiency values below 20%.

#### Establishment of the LY6E-KO cell line

CRISPR-Cas9–mediated knockout of *LY6E* was performed using a lentiviral delivery system. A guide RNA (gRNA) was selected using CRISPRdirect (https://crispr.dbcls.jp/) with the following sequence.

**Table:** 

The following gRNA sequences (5′-3′) were used for CRISPR-Cas9 genome editing:
GCCCTTCTGGGTGTGGAGCG (PAM: AGG)

The gRNA was inserted into the U6-sgRNA backbone derived from AAV:ITR-U6-sgRNA(backbone)-pCBh-Cre-WPRE-hGHpA-ITR (Addgene plasmid #60229). For lentiviral backbone construction, Lenti-Cas9-blast (Addgene plasmid #52962) was modified by inserting mCherry between the BamHI and EcoRV restriction recognition sites, generating Lenti-Cas9-mCherry. Subsequently, the U6-sgRNA cassette was introduced at the upstream NheI site of the Cas9-mCherry expression cassette, yielding Lenti-U6-gRNA-Cas9-mCherry. Lentiviral particles were produced from this vector and applied to HEK293T cells at a multiplicity of infection corresponding to 1. One week after infection, mCherry-positive cells were single-cell sorted using a Cell Sorter MA900 (Sony). Clonal populations were expanded, and genomic DNA was extracted using QIAamp DNA Mini Kit (QIAGEN). The genomic region flanking the gRNA target site was amplified by PCR and subjected to Sanger sequencing for genotyping, confirming frameshift mutations in *LY6E*.

#### Cell culture for AAV infectivity evaluation

HEK293T cells were seeded at 80% confluency in 96-well plates and maintained in DMEM supplemented with 5% FBS, and PS (100 U/mL) at 37°C in 5% CO_2_. Target candidates (150 ng) were transiently expressed in HEK293T cells. DNA transfection was performed using Lipofectamine 3000 according to the manufacturer’s instructions (Invitrogen). Cells expressing each target candidate were transduced with engineered AAV variants at 5 × 10^9^ v.g. per well in triplicate 24 h after transfection. Receptor-expressing cells were transferred to 96-well plates at 20% confluency and maintained in FluoroBrite DMEM supplemented with 0.5% FBS, 1% NEAA, PS (100 U/mL) at 37°C in 5% CO_2_. Images were acquired with a fluorescence microscope (Axio Observer 7, ZEISS) and processed using ZEN 3.4 (Zeiss) and ImageJ software.

#### Cell culture fluorescence image quantitation

All image analyses were performed using a custom Python-based image processing pipeline developed for automated quantification of transduction efficiency and fluorescence intensity. The pipeline processed paired bright-field and fluorescence images, extracting metrics including total cell area, transduced area, transduction percentage, and fluorescence intensity per transduced area. For both image types, background subtraction was performed by first converting RGB images to grayscale, followed by Gaussian blurring (skimage.filters.Gaussian) and subtracting the blurred image from the original. For bright-field images, a Gaussian filter with sigma = 30 and truncate = 0.35 was applied. Histogram-based thresholding (skimage.filters.threshold_otsu) was then used on both the subtracted image and its inverted version to detect bright and dark regions corresponding to cell edges. These binary masks were combined and morphologically closed (skimage.morphology. closing with a disk of radius 2) to fill in cellular regions. The total cell area was determined by summing the pixels in this final mask. For fluorescence signal images, a Gaussian filter with sigma = 100 and truncate = 0.35 was applied, and the resulting blurred image was subtracted from the original to remove background fluctuations. Histogram-based thresholding was used to identify regions with strong fluorescence, corresponding to transduced cells. Small noise artifacts were removed using skimage. morphology.remove_small_objects (minimum size = 5). The transduced area was calculated as the total number of pixels in this cleaned mask. To quantify signal intensity, the fluorescence image was multiplied by the binary mask of transduced cells, and the resulting pixel intensities were summed to obtain the total brightness of transduced regions. This value was then normalized by transduced area to yield the brightness per transduced area. To avoid overestimation in cases with extremely low signal or cell area, a lower threshold of 0.1% was enforced for the transduction percentage.

Paired bright-field and fluorescence images were automatically matched and analyzed from a specified folder. The image filenames were parsed to extract metadata such as serotype and dose. All calculated values were stored in a summary CSV file using the pandas library.

#### Fluorescence staining and image-based quantification of double-positive cells

To analyze the distribution of cells co-expressing two molecular markers, brain sections from the thalamus, cortex, and hippocampus were subjected to fluorescent staining and quantitative image analysis. Coronal brain sections of 30 μm thickness were mounted onto glass slides and air-dried overnight at room temperature. Sections were then briefly washed in PBS for 5 min, followed by a 10 min wash in PBST (PBS containing 0.1% Triton X-100), and two additional 5 min washes in PBS. Staining was performed by applying 200 μL of the 1/20 diluted NeuroTrace 530/615 Red Fluorescent Nissl Stain (Thermo Fisher Scientific) solution to each section and incubating at room temperature for 20 min. After staining, sections were washed again in PBST for 10 min and twice more in PBS (2 × 5 min). A final wash in PBS was performed either for 2 h at room temperature or overnight at 4°C. After washing, sections were coverslipped and stored at 4°C until imaging. Fluorescent images of each region were acquired using a fluorescence microscope. For each sample, two grayscale images representing different fluorescent markers were exported as 16-bit TIFF files. These images were analyzed using a custom Python script based on OpenCV, scikit-image, and matplotlib libraries. Each image was converted to grayscale if it contained RGB information, then binarized using fixed intensity thresholds selected empirically to minimize background noise while retaining cellular signals. In the representative analysis, a threshold of 100 was applied to the first image (channel 1) and a threshold of 10 to the second image (channel 2). Binarized images were processed using an 8-connected component labeling algorithm to identify signal-positive regions. These labeled regions, corresponding to individual cells, were characterized using morphological feature extraction. To assess signal co-localization, each cell identified in the first image was examined for pixel-wise overlap with the labeled regions in the second image. A cell was classified as double-positive if at least one of its pixels overlapped with a region in the second channel. The proportion of double-positive cells was calculated as the number of overlapping cells divided by the total number of cells in the channel that contained fewer cells. This procedure prevented overestimation due to differences in segmentation sensitivity between channels. The results were visualized as pie charts, with double-positive cells categorized as “Neuron” and the remaining single-positive cells as “Glia.” All analyses and visualizations were saved as image files corresponding to the specific brain regions.

#### Structural analyses

Peptide–receptor structures were modeled using AlphaFold-Multimer, which is based on AlphaFold3.^29^^,^^37^ As the query sequence, we used the extracellular domain of Hamster Ly6e (UniProt ID: A0A1U7Q6V5_MESAU), as described earlier. All other parameters were kept at their default settings. AAV trimer–receptor complex models were constructed using a hybrid structural modeling approach inspired by the strategy described in Shay et al., 2023.^40^ Trimers located at the AAV 3-fold symmetry axis were selected as the minimal binding interface that could approximate interactions between the full capsid and the putative receptor while maintaining computational tractability.

First, a peptide–receptor complex was modeled by inputting a 15-amino-acid peptide (residues 587–594 based on WT AAV9 VP1 numbering) from the AAV.Cap-PF1.7 variant together with the target receptor, as described above. Separately, a trimer model of the AAV.Cap-PF1.7 was generated using AlphaFold-Multimer. From the peptide–receptor model, the two residues with the highest predicted confidence (pLDDT score) Trp1′ and Met2′ were structurally aligned to the corresponding residues on the first subunit of the trimer model (coarse combined model). The two loops between Ala587 and Ala589 (VP1 indices) were remodeled using RosettaRemodel^46^ from the Rosetta software bundle (release 2018.48.60516). Lastly, these remodeled loops were merged to generate the final model. pLDDT scores for individual residues from the original AlphaFold-Multimer outputs were used to color images of the final model to generate a visual representation of predicted confidence. The final model was further optimized using the Rosetta fast-relax protocol.

#### RT-qPCR analysis of hamster brain

Total RNA was isolated from hamster brain tissue using the RNeasy Mini Kit (QIAGEN) according to the manufacturer’s instructions. Approximately 20–30 mg of brain tissue was rapidly frozen in liquid nitrogen, pulverized to a fine powder, and subsequently homogenized with a Microtube Homogenizer G10 (Bio Medical Science Inc.). cDNA was synthesized from total RNA using a QuantiTect Reverse Transcription Kit (QIAGEN), following the manufacturer’s protocol. *Gapdh* was used as the internal control. All oligonucleotide primer sequences used for qPCR are given in Table S1. Quantitative PCR was performed with TB Green Premix Ex Taq GC (Perfect Real Time, TaKaRa Bio Inc) under the following conditions: initial denaturation at 95°C for 60 s, followed by 45 cycles of denaturation at 95°C for 10 s and annealing/extension at 60°C for 30 s. Relative gene expression levels were calculated using the 2^−ΔCt^ method, normalized to *Gapdh*. Primer amplification efficiencies were evaluated from the raw amplification curves (ΔRn) by estimating reaction efficiency in the log-linear phase. Because efficiencies for the Ly6 primer sets were generally high (≥90% for most targets), no additional efficiency-based correction was applied to the reported relative expression values. Data are grouped by gene. Mean ± standard deviation (SD) values are reported.

### Quantification and statistical analysis

Data representation and statistical analyses were performed in Python. One-way ANOVA followed by Tukey’s post hoc test was used in the analysis of RT-qPCR data. Statistical significance is indicated as follows: *p* < 0.05 (∗), *p* < 0.01 (∗∗), *p* < 0.001 (∗∗∗), and *p* < 0.0001 (∗∗∗∗). For comparisons between *Ly6e* and other genes within each brain region, the Mann–Whitney U test was used, followed by multiple testing correction with the Benjamini–Hochberg method. Statistical significance is indicated as follows: *p* < 0.05 (∗), *p* < 0.01 (∗∗), *p* < 0.001 (∗∗∗). For statistical testing of *in vitro* potency assays and image-based analyses, Welch’s two-sided *t* test was used, with Holm–Bonferroni correction applied for multiple comparisons where appropriate. Statistical significance is indicated as follows: *p* < 0.05 (∗), *p* < 0.01 (∗∗), *p* < 0.001 (∗∗∗), and *p* < 0.0001 (∗∗∗∗). For *in vivo* comparisons of transduction across capsids within each brain region, Welch’s two-sided *t* test was used. In figures where daggers are used, † indicates *p* < 0.05 and †† indicates *p* < 0.01. *ns* indicates not significant. Exact statistical tests, multiple-comparison procedures, sample sizes, and definitions of data presentation are provided in the corresponding figure legends.
