# Supplementary material for: Computationally guided discovery of Ly6e/LY6E-dependent AAV capsid variants
Source: iScience. 2026 Apr 1;29(5):115554. doi: 10.1016/j.isci.2026.115554 (PMC13101293; doi:10.1016/j.isci.2026.115554)
Supplement: Document S1. Figures S1–S5 and Table S1 [file mmc1.pdf]

iScience, Volume *29*

Supplemental information

Computationally guided discovery of Ly6e/LY6E-dependent AAV capsid variants

Hiroaki Ono, Shoko Fujino, and Genshiro A. Sunagawa

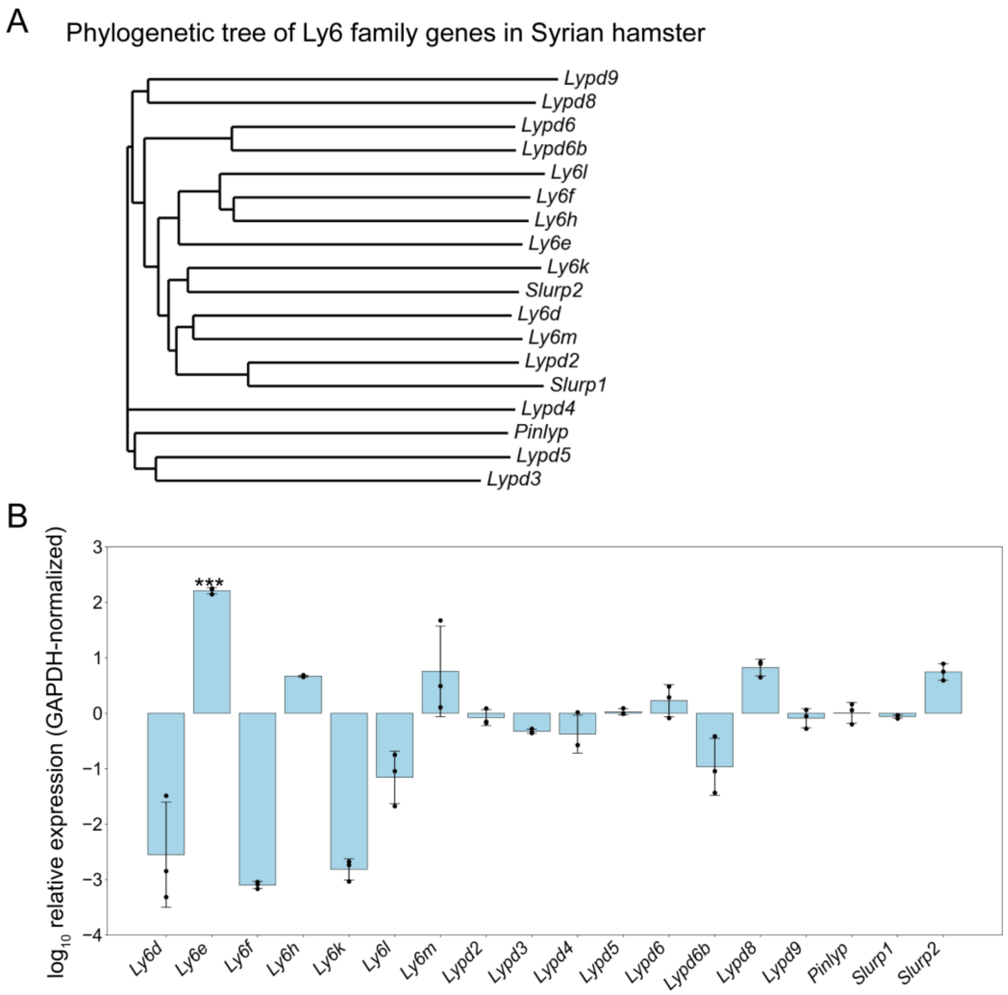

Figure S1 RT-qPCR analysis of Ly6 family gene expression in hamster brain

(A) Ly6 family genes were manually extracted from the annotated genes in the golden hamster genome (Baylor, 2021) registered in the genome browser. A phylogenetic tree was constructed using Clustal Omega. (B) Log_{10}-transformed relative expression values of each Ly6 family gene (normalized to *Gapdh*) are shown. Data represent mean ± SD of n = 3 technical replicates. One-way ANOVA followed by Tukey's post hoc test was used. Asterisks indicate significance relative to the indicated comparisons (*$p < 0.05$, **$p < 0.01$, ***$p < 0.001$).

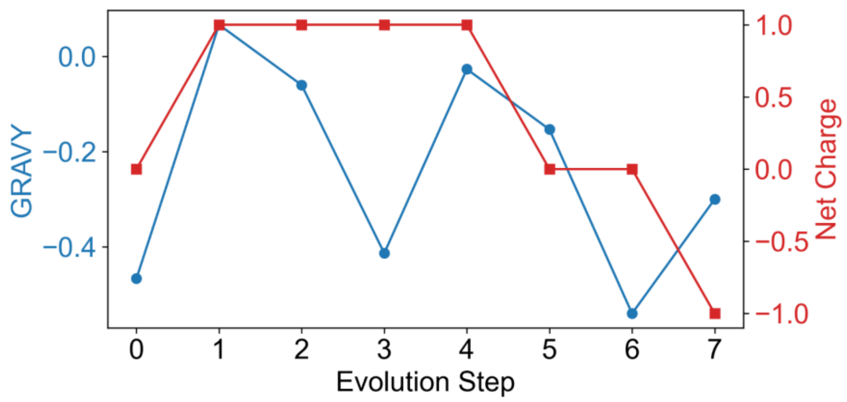

Figure S2 Cyclic changes in hydropathy and charge during sequence evolution

Average hydropathy (GRAVY score; blue line, left y-axis) and net charge (red line, right y-axis) were calculated for each sequence across the evolutionary steps. Hydropathy values were obtained by averaging Kyte–Doolittle indices across all residues in a sequence, whereas net charge was estimated at pH 7 by assigning Asp/Glu = –1, Lys/Arg = +1, and His = +0.1. A recurring pattern was observed in which an increase in sequence hydropathy was followed by compensatory adjustments in net charge during the evolutionary process.

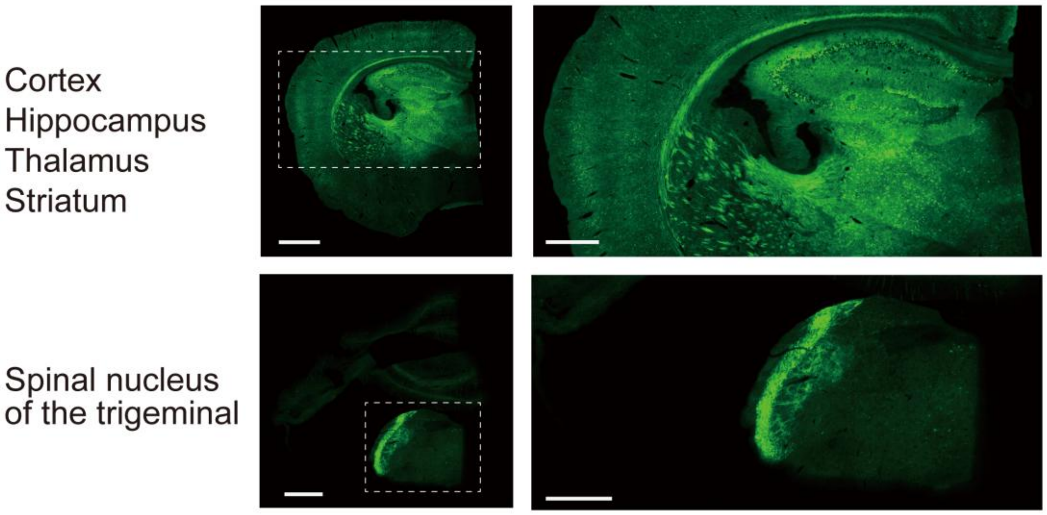

Figure S3 CNS tropism of AAV capsids in Syrian hamsters

Representative sections from Cap-PF1.7–injected animals: a coronal brain section at the striatal level and a transverse section at the dorsal spinal cord level. Scale bars, 3 mm (sagittal and coronal image) and 500 μm (brain region).

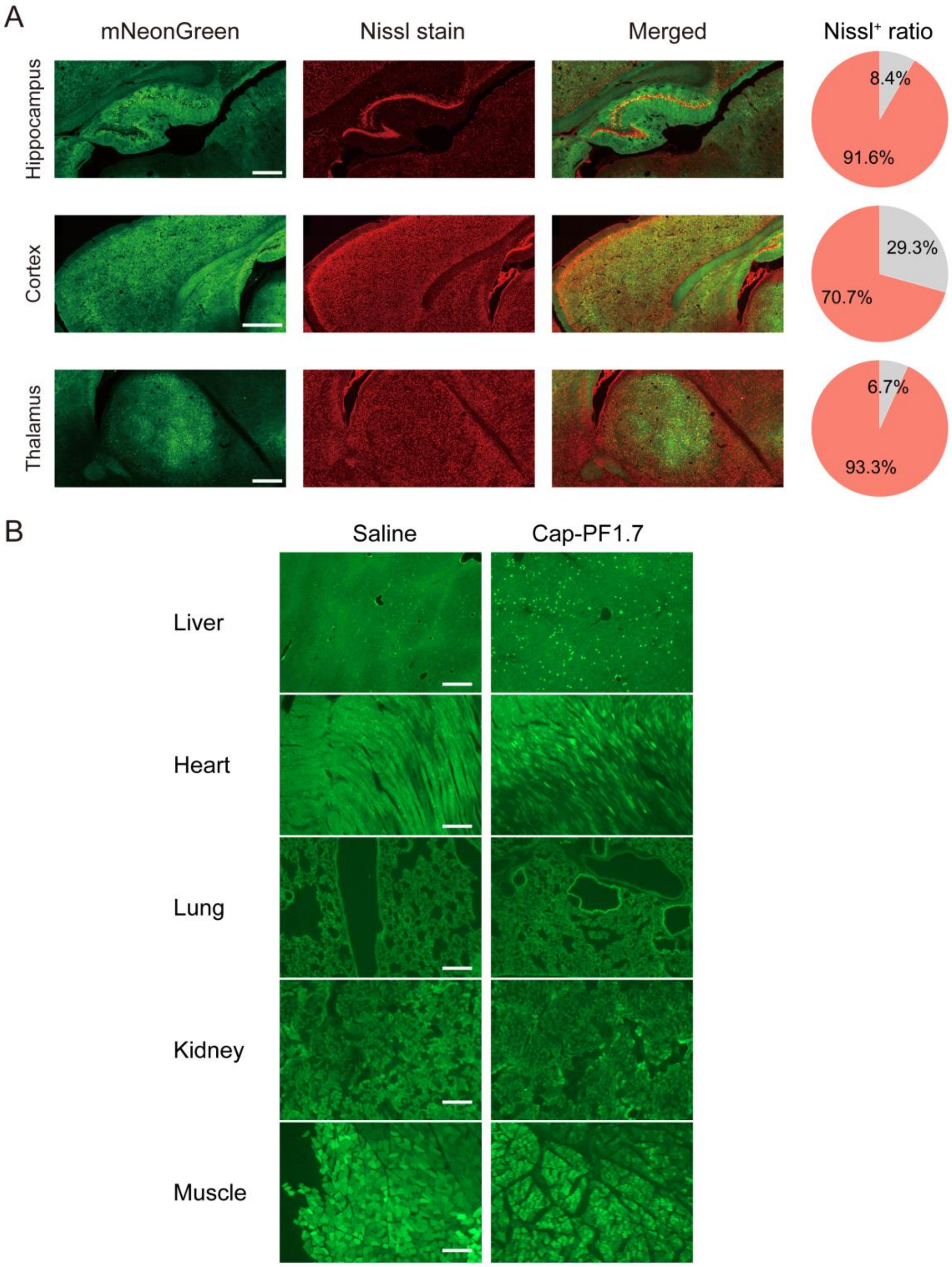

A
mNeonGreen
Nissl stain
Merged
Nissl+ ratio
Hippocampus
8.4%
91.6%
Cortex
29.3%
70.7%
Thalamus
6.7%
93.3%
B
Saline
Cap-PF1.7
Liver
Heart
Lung
Kidney
Muscle

Figure S4

Figure S4 Cap-PF1.7 targeting cell type and off-target tissue validation

(A) NeuroTrace assessment of representative brain regions to evaluate the cell types targeted by Cap-PF1.7. Colocalization of mNeonGreen fluorescence (driven by the CAG promoter) and NeuroTrace signal were assessed for each brain section. Pie charts indicate the proportion of NeuroTrace-positive cells among mNeonGreen-expressing cells. AAVs were administered intravenously to Syrian hamsters at a dose of 1×10^{12} v.g. per animal (n = 3 per condition). Four weeks after administration, transgene expression was assessed by visualizing mNeonGreen fluorescence throughout the brain. Scale bars, 500 μm. (B) Evaluation of mNeonGreen fluorescence (driven by the CAG promoter) in peripheral tissues. AAVs were administered intravenously to Syrian hamsters at a dose of 1×10^{12} v.g. per animal (n = 3 per condition). Four weeks after administration, transgene expression was assessed by mNeonGreen fluorescence. Scale bars, 500 μm.

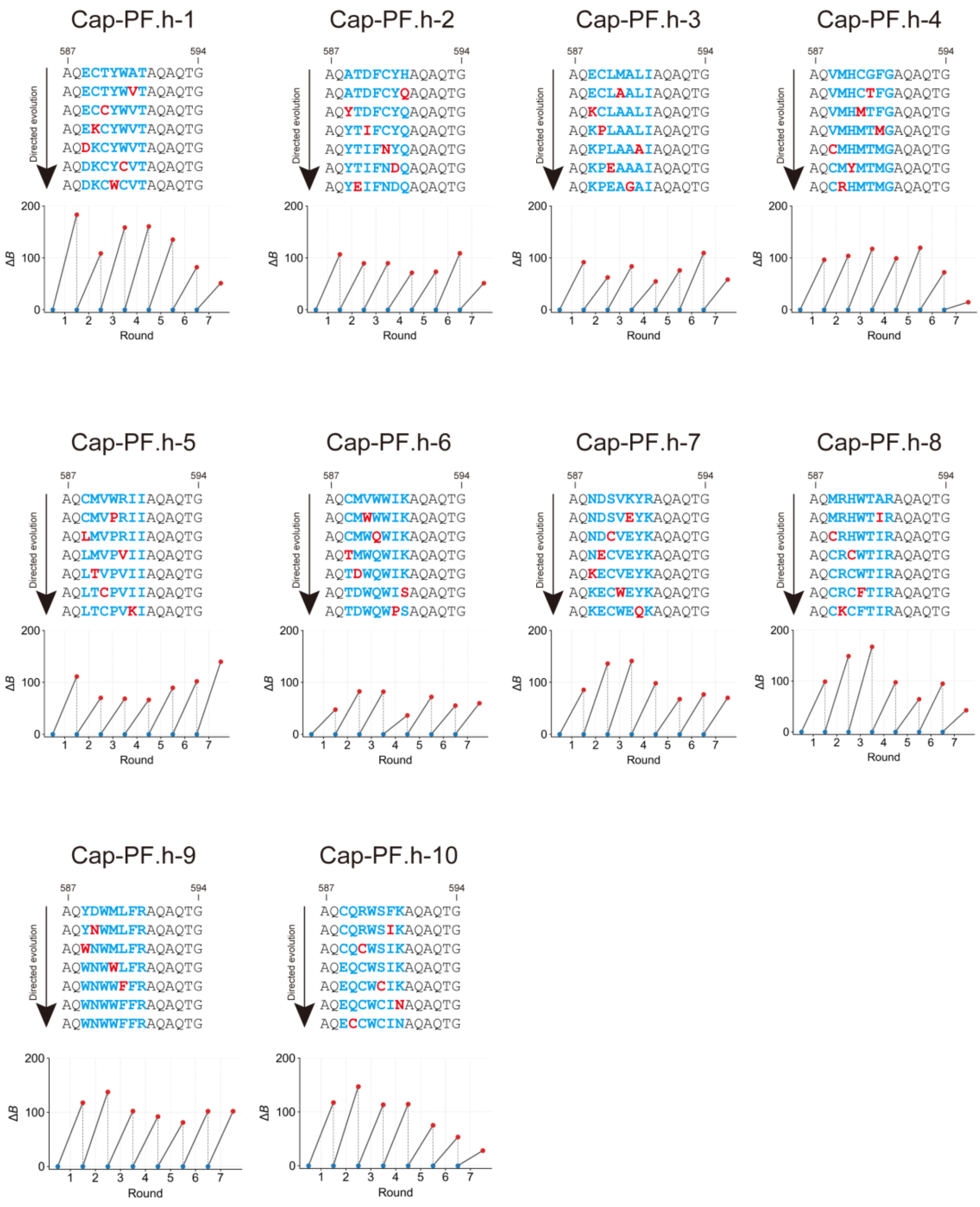
Cap-PF.h-1
587
594
AQECTYWATAQAQTG
AQECTYWVTAQAQTG
AQECCYWVTAQAQTG
AQEKCYWVTAQAQTG
AQDKCYWVTAQAQTG
AQDKCYCVTAQAQTG
AQDKCWCVTAQAQTG
Directed evolution
ΔB
Round
Cap-PF.h-2
AQATDFCYHAQAQTG
AQATDFCYQAQAQTG
AQYTDFCYQAQAQTG
AQYTIFCYQAQAQTG
AQYTIFNYQAQAQTG
AQYTIFNDQAQAQTG
AQYEIFNDQAQAQTG
Cap-PF.h-3
AQECLMALIAQAQTG
AQECLAALIAQAQTG
AQKCLAALIAQAQTG
AQKPLAALIAQAQTG
AQKPLAAAIAQAQTG
AQKPEAAAIAQAQTG
AQKPEAGAIAQAQTG
Cap-PF.h-4
AQVMHCGFGAQAQTG
AQVMHCTFGAQAQTG
AQVMHMTFGAQAQTG
AQVMHMTMGAQAQTG
AQCMHMTMGAQAQTG
AQCMYMTMGAQAQTG
AQCRHMTMGAQAQTG
Cap-PF.h-5
AQCMVWRIIAQAQTG
AQCMVPRIIAQAQTG
AQLMVPRIIAQAQTG
AQLMVPVIIAQAQTG
AQLTVPVIIAQAQTG
AQLTCPVIIAQAQTG
AQLTCPVKIAQAQTG
Cap-PF.h-6
AQCMVWWIKAQAQTG
AQCMWWWIKAQAQTG
AQCMWQWIKAQAQTG
AQTMWQWIKAQAQTG
AQTDWQWIKAQAQTG
AQTDWQWISAQAQTG
AQTDWQWPSAQAQTG
Cap-PF.h-7
AQNDSVKYRAQAQTG
AQNDSVEYKAQAQTG
AQNDCVEYKAQAQTG
AQNECVEYKAQAQTG
AQKECVEYKAQAQTG
AQKECWEYKAQAQTG
AQKECWEQKAQAQTG
Cap-PF.h-8
AQMRHWTARAQAQTG
AQMRHWTIRAQAQTG
AQCRHWTIRAQAQTG
AQCRCWTIRAQAQTG
AQCRCWTIRAQAQTG
AQCRCFTIRAQAQTG
AQCKCFTIRAQAQTG
Cap-PF.h-9
AQYDWMLFRAQAQTG
AQYNWMLFRAQAQTG
AQWNWMLFRAQAQTG
AQWNWWLFRAQAQTG
AQWNWWFFRAQAQTG
AQWNWWFFRAQAQTG
AQWNWWFFRAQAQTG
Cap-PF.h-10
AQCQRWSFKAQAQTG
AQCQRWSIKAQAQTG
AQCQCWSIKAQAQTG
AQEQCWSIKAQAQTG
AQEQCWCIKAQAQTG
AQEQCWCINAQAQTG
AQECCWCINAQAQTG

Figure S5

Figure S5 Directed-evolution trajectory of LY6E-binding peptides

For each lineage, the amino-acid sequences of the capsid-displayed 7-mer peptide inserted at the AAV VP1 peptide-insertion site (residues 588–594; shown in blue, flanked by VP1 residues 587 and 594) are listed for each round of selection (arrow). Red letters indicate amino-acid substitutions that arose in that round relative to the preceding variant. The plots beneath each lineage summarize the round-by-round change in the functional score (ΔB) during directed evolution; points indicate the ΔB measured for each round, and connecting lines denote progression across rounds (the red point corresponds to the variant carried forward to the next round).

Table S1. Sequences of oligonucleotides used for plasmid construction, RT-qPCR and cloning

No	Name	Sequence	Note
1	Fwd_PF1.0	CCTACATCATCGCACAGGCGCAGACCGGCTG	Forward PCR primer for inserting Cap-PF1.0 into the AAV9 capsid
2	Rev_PF1.0	CGGGCAGTCTTTGGGCACTCTGGTGGTTTG	Reverse PCR primer for inserting Cap-PF1.0 into the AAV9 capsid
3	Fwd_PF1.1	CCTACATCATCGCACAGGCGCAGACCGGTTG	Forward PCR primer for inserting Cap-PF1.1 into the AAV9 capsid
4	Rev_PF1.1	CGGGCAGTCTTTGGGCACTCTGGTGGTTTG	Reverse PCR primer for inserting Cap-PF1.1 into the AAV9 capsid
5	Fwd_PF1.2	CCTACATCATCGCACAGGCGCAGACCGGTTG	Forward PCR primer for inserting Cap-PF1.2 into the AAV9 capsid
6	Rev_PF1.2	CGGGCATTCTTTGGGCACTCTGGTGGTTTG	Reverse PCR primer for inserting Cap-PF1.2 into the AAV9 capsid
7	Fwd_PF1.3	AGTACATCATCGCACAGGCGCAGACCGGTTG	Forward PCR primer for inserting Cap-PF1.3 into the AAV9 capsid
8	Rev_PF1.3	GGGGCATTCTTTGGGCACTCTGGTGGTTTG	Reverse PCR primer for inserting Cap-PF1.3 into the AAV9 capsid
9	Fwd_PF1.4	AGATCATCATCGCACAGGCGCAGACCGGTTG	Forward PCR primer for inserting Cap-PF1.4 into the AAV9 capsid
10	Rev_PF1.4	GGGGCATTCTTTGGGCACTCTGGTGGTTTG	Reverse PCR primer for inserting Cap-PF1.4 into the AAV9 capsid
11	Fwd_PF1.5	AGATCATCATCGCACAGGCGCAGACCGGTTG	Forward PCR primer for inserting Cap-PF1.5 into the AAV9 capsid
12	Rev_PF1.5	GGTCCATTCTTTGGGCACTCTGGTGGTTTG	Reverse PCR primer for inserting Cap-PF1.5 into the AAV9 capsid
13	Fwd_PF1.6	AGATCATCTACGCACAGGCGCAGACCGGTTG	Forward PCR primer for inserting Cap-PF1.6 into the AAV9 capsid
14	Rev_PF1.6	GGTCCATTCTTTGGGCACTCTGGTGGTTTG	Reverse PCR primer for inserting Cap-PF1.6 into the AAV9 capsid
15	Fwd_PF1.7	AGATCATCTACGCACAGGCGCAGACCGGCTG	Forward PCR primer for inserting Cap-PF1.7 into the AAV9 capsid
16	Rev_PF1.7	GGTCCATCCATTGGGCACTCTGGTGGTTTG	Reverse PCR primer for inserting Cap-PF1.7 into the AAV9 capsid
17	Fwd_PF.h1.1	GGTGCGTGACCGCACAGGCGCAGACCGGCTG	Forward PCR primer for inserting Cap-PF.h1.1 into the AAV9 capsid
18	Rev_PF.h1.1	AGCACTTGTCTTGGGCACTCTGGTGGTTTG	Reverse PCR primer for inserting Cap-PF.h1.1 into the AAV9 capsid
19	Fwd_PF.h1.2	TCAACGACCAGGCACAGGCGCAGACCGGCTG	Forward PCR primer for inserting Cap-PF.h1.2 into the AAV9 capsid
20	Rev_PF.h1.2	AGATCTCGTATTGGGCACTCTGGTGGTTTG	Reverse PCR primer for inserting Cap-PF.h1.2 into the AAV9 capsid
21	Fwd_PF.h1.3	CCGGCGCCATTGCACAGGCGCAGACCGGCTG	Forward PCR primer for inserting Cap-PF.h1.3 into the AAV9 capsid
22	Rev_PF.h1.3	CCTCAGGTTTTTGGGCACTCTGGTGGTTTG	Reverse PCR primer for inserting Cap-PF.h1.3 into the AAV9 capsid
23	Fwd_PF.h1.4	TGACCATGGGCGCACAGGCGCAGACCGGCTG	Forward PCR primer for inserting Cap-PF.h1.4 into the AAV9 capsid
24	Rev_PF.h1.4	TGTGTCTGCATTGGGCACTCTGGTGGTTTG	Reverse PCR primer for inserting Cap-PF.h1.4 into the AAV9 capsid
25	Fwd_PF.h1.5	CCGTGAAGATCGCACAGGCGCAGACCGGCTG	Forward PCR primer for inserting Cap-PF.h1.5 into the AAV9 capsid
26	Rev_PF.h1.5	GGCAGGTCAGTTGGGCACTCTGGTGGTTTG	Reverse PCR primer for inserting Cap-PF.h1.5 into the AAV9 capsid
27	Fwd_PF.h1.6	AGTGGCCTAGCGCACAGGCGCAGACCGGCTG	Forward PCR primer for inserting Cap-PF.h1.6 into the AAV9 capsid
28	Rev_PF.h1.6	GCCAATCGGTTTGGGCACTCTGGTGGTTTG	Reverse PCR primer for inserting Cap-PF.h1.6 into the AAV9 capsid
29	Fwd_PF.h1.7	GGGAGCAGAAGGCACAGGCGCAGACCGGCTG	Forward PCR primer for inserting Cap-PF.h1.7 into the AAV9 capsid
30	Rev_PF.h1.7	AGCACTCCTTTTGGGCACTCTGGTGGTTTG	Reverse PCR primer for inserting Cap-PF.h1.7 into the AAV9 capsid
31	Fwd_PF.h1.8	TCACCATCAGAGCACAGGCGCAGACCGGCTG	Forward PCR primer for inserting Cap-PF.h1.8 into the AAV9 capsid
32	Rev_PF.h1.8	AGCACTTGCATTGGGCACTCTGGTGGTTTG	Reverse PCR primer for inserting Cap-PF.h1.8 into the AAV9 capsid
33	Fwd_PF.h1.9	GGTTCTTCAGAGCACAGGCGCAGACCGGCTG	Forward PCR primer for inserting Cap-PF.h1.9 into the AAV9 capsid
34	Rev_PF.h1.9	ACCAGTTCCATTGGGCACTCTGGTGGTTTG	Reverse PCR primer for inserting Cap-PF.h1.9 into the AAV9 capsid
35	Fwd_PF.h1.10	GGTGCATCAACGCACAGGCGCAGACCGGCTG	Forward PCR primer for inserting Cap-PF.h1.10 into the AAV9 capsid
36	Rev_PF.h1.10	AGCAGCACTCTTGGGCACTCTGGTGGTTTG	Reverse PCR primer for inserting Cap-PF.h1.10 into the AAV9 capsid
37	Fwd_LY6E_gRNA	GTGTGGAGCGGTTTTAGAGCTAGAAATAGCAAG	Forward PCR primer to synthesize LY6E_gRNA
38	Rev_LY6E_gRNA	CCAGAAGGGCGGTGTTTCGTCCTTTCCACAAG	Reverse PCR primer to synthesize LY6E_gRNA
39	Gapdh_qPCR_F	TCTCTATCCCTTCTTCTGATGC	Forward qPCR primer for Gapdh
40	Gapdh_qPCR_R	GGACCATCTACAGTCTTCTGTGTG	Reverse qPCR primer for Gapdh
41	Ly6d_qPCR_F	TGAAGACGGCTCTGCTACTCC	Forward qPCR primer for Ly6d
42	Ly6d_qPCR_R	GTTGTTTACAGTTCGCGCTG	Reverse qPCR primer for Ly6d
43	Ly6e_qPCR_F	GTCAACTTAGGTGTGGCATCTG	Forward qPCR primer for Ly6e
44	Ly6e_qPCR_R	CAAGCTGAGCAGGAGTCCAAGG	Reverse qPCR primer for Ly6e
45	Ly6f_qPCR_F	GAACAGTTCCCATGCTATGAAG	Forward qPCR primer for Ly6f
46	Ly6f_qPCR_R	CTTGCTGATGATGGTGATACCCGG	Reverse qPCR primer for Ly6f
47	Ly6h_qPCR_F	TTTGTGCCAGCGTGCGGATC	Forward qPCR primer for Ly6h
48	Ly6h_qPCR_R	CACGAAGTCGCAGGAGGAAG	Reverse qPCR primer for Ly6h
49	Ly6k_qPCR_F	CAGCAGATTCTCAGACCTCG	Forward qPCR primer for Ly6k
50	Ly6k_qPCR_R	CTCTGCCTCACAGACGTGAC	Reverse qPCR primer for Ly6k
51	Ly6l_qPCR_F	AGCCCATAAAGTGCCAGCCAG	Forward qPCR primer for Ly6l
52	Ly6l_qPCR_R	CTGTTGGTGTTGGGACACTGGAC	Reverse qPCR primer for Ly6l
53	Ly6m_qPCR_F	CGCTACTGTCTGACCTCCTG	Forward qPCR primer for Ly6m
54	Ly6m_qPCR_R	GGAGTTGTTCTGGCTCTGATGG	Reverse qPCR primer for Ly6m
55	Lypd2_qPCR_F	TCATCTTGGAACACCTGCTG	Forward qPCR primer for Lypd2
56	Lypd2_qPCR_R	GTGACCGTTGTACTTCTTCAAGTC	Reverse qPCR primer for Lypd2
57	Lypd3_qPCR_F	GGGTAGGTTACTCATCTGAGG	Forward qPCR primer for Lypd3
58	Lypd3_qPCR_R	CACATCTCCTCTCCTGGCAG	Reverse qPCR primer for Lypd3
59	Lypd4_qPCR_F	TCACATCCAGGACCCTGACAC	Forward qPCR primer for Lypd4
60	Lypd4_qPCR_R	CTAGGCAATAAAGAGACGGCC	Reverse qPCR primer for Lypd4
61	Lypd5_qPCR_F	CATCAGAATTTGGCTCCAGAC	Forward qPCR primer for Lypd5
62	Lypd5_qPCR_R	CTAGGCAATAAAGAGACGGCC	Reverse qPCR primer for Lypd5
63	Lypd6_qPCR_F	CTTTGAGGCCCTCTGTATGAGTG	Forward qPCR primer for Lypd6

Table S1. Sequences of oligonucleotides used for plasmid construction, RT-qPCR and cloning (Continued)

64	Lypd6_qPCR_R	GGATTGAGCAGCTTTCAGACAGTC	Reverse qPCR primer for Lypd6
65	Lypd6b_qPCR_F	CACAGAAGCCCAGTGAGTGC	Forward qPCR primer for Lypd6b
66	Lypd6b_qPCR_R	CTGGTAGTCGGGCTTTGCTCTGC	Reverse qPCR primer for Lypd6b
67	Lypd8_qPCR_F	CCATCCTTAGGAGAAGTCAGTC	Forward qPCR primer for Lypd8
68	Lypd8_qPCR_R	CCATCGCCACATTCTGAAGC	Reverse qPCR primer for Lypd8
69	Lypd9_qPCR_F	ACTGAGTACATCTTTGGCTGTC	Forward qPCR primer for Lypd9
70	Lypd9_qPCR_R	GACTCCTGGGCTTCACATGC	Reverse qPCR primer for Lypd9
71	Pinlyp_qPCR_F	GGCTTCCTATAAGGGCACAG	Forward qPCR primer for Pinlyp
72	Pinlyp_qPCR_R	GTGTGAGAGCCAGCAGGAAGGTC	Reverse qPCR primer for Pinlyp
73	Slurp1_qPCR_F	CAAACATGAAACCAGGCAGGCTC	Forward qPCR primer for Slurp1
74	Slurp1_qPCR_R	CCATGTTCCAGGCTGCCAAG	Reverse qPCR primer for Slurp1
75	Slurp2_qPCR_F	TCCTCAAACATGAAACCAGGCAG	Forward qPCR primer for Slurp2
76	Slurp2_qPCR_R	CCATGTTCCAGGCTGCCAAG	Reverse qPCR primer for Slurp2
